# Detectability of a poison frog and its Batesian mimic depends on body posture and viewing angle

**DOI:** 10.1093/beheco/arae077

**Published:** 2024-10-04

**Authors:** Brendan L McEwen, Justin Yeager, Isaac Kinley, Hannah M Anderson, James B Barnett

**Affiliations:** Department of Psychology, Neuroscience, & Behaviour, McMaster University, Hamilton, ON L8S 4L8, Canada; Grupo de Investigación en Biodiversidad, Medio Ambiente y Salud (BIOMAS), Facultad de Ingenierías y Ciencas Aplicadas, Universidad de Las Américas, Ecuador; Department of Psychology, Neuroscience, & Behaviour, McMaster University, Hamilton, ON L8S 4L8, Canada; Rotman Research Institute at Baycrest, Toronto, ON M6A 1W1, Canada; Department of Psychology, Neuroscience, & Behaviour, McMaster University, Hamilton, ON L8S 4L8, Canada; Department of Psychology, Neuroscience, & Behaviour, McMaster University, Hamilton, ON L8S 4L8, Canada; School of Natural Sciences, Trinity College Dublin, Dublin 2 D02 PN40, Ireland

**Keywords:** Aposematism, Batesian mimicry, detectability, imperfect mimicry, poison frogs, visual ecology

## Abstract

Aposematic signals warn predators that prey should be avoided due to dangerous secondary defences. However, as warning signals do not always produce avoidance, warning colors may evolve as a trade-off balancing detectability against signal saliency. For Batesian mimics, which display salient signals but lack secondary defenses, the costs of predator encounters are greater, potentially increasing the benefit of crypsis. This raises the question of whether imperfect mimicry may reduce detectability while retaining mimetic efficacy. We tested this hypothesis with the poisonous frog *Ameerega bilinguis* and undefended Batesian mimic *Allobates zaparo,* using computational visual modeling and screen-based detection trials with human participants. We found that both species incorporate camouflage into their warning colors, but to different degrees depending on viewing angle and behavior. Contrary to expectation, we found differences in detectability between model and mimic that do not adhere to the hypothesized cryptic mimetic phenotype. To aerial observers, we found the mimic to be more detectable than the model. To terrestrial observers, likely owing to the model’s bright ventral color, we found the model more detectable in viewing angles that highlight the ventral coloration, whereas the mimic was more detectable in viewing angles that highlight the dorsal coloration. Consequently, we suggest that in addition to being the result of perceptual or developmental constraints, imperfect mimicry may also evolve as an adaptive strategy which balances camouflage with different signaling functions. Our findings complement the emerging view that aposematic signals may evolve in response to a multitude of selection pressures beyond aversion alone.

## Teaser

We tested the hypothesis that mimics should produce a less-detectable version of their model’s coloration in poison frogs. Surprisingly, we found the mimic to be the more detectable species. Why is this surprising? Toxic species evolve bright coloration to warn predators of their chemical defense. Nontoxic species may mimic these colors, deceiving predators and reducing their own risk of attack. However, as predators may see through this disguise, being too conspicuous can be risky. Consequently, it has been suggested that mimics should produce a less-detectable version of their model’s coloration. We showed that this is not always the case.

## Introduction

The natural world teems with predation threats that impose strong selection on prey, and a diverse array of antipredator strategies have subsequently evolved in response (Cuthill et al. 2017; Ruxton et al. 2018). One such strategy is aposematism, in which prey produce defenses (e.g. noxious chemical compounds) and evolve visual cues that advertise their unprofitability to potential predators (Stevens and Ruxton 2012). Predators learn and/or evolve to avoid prey based on these signals, allowing aposematic species to exploit their environment while reducing the opportunity costs imposed by the need for cryptic behavior (Skelhorn et al. 2016).

As predators will not always avoid aposematic prey, aposematic signals may instead be considered a reconciliation of (1) the benefits of a low predator encounter rate through camouflage and (2) the benefits of increasingly distinctive and salient warning colors (Endler and Mappes 2004; Stevens 2007). Consequently, even when selection appears to primarily favor aposematism, animal coloration can continue to serve multiple purposes. Salient signals may therefore be under simultaneous selection for functions including crypsis and intraspecific communication, in addition to aposematism (Maan and Cummings 2008, 2009; Cuthill et al. 2017; Kikuchi et al. 2023). Where selection is complementary conspicuous colors may be co-opted and exaggerated through both natural and sexual selection, whereas antagonistic selection pressures may necessitate trade-offs and compromise (Postema et al. 2022; Yeager and Penacchio 2023). Alternatively, opposing selection may also favor the evolution of multicomponent signals, where different patches convey different information, and multifunctional signals where a single patch acts in different ways depending on context (Postema et al. 2022). These multipurpose phenotypes are suggested to reduce the costs imposed by conflicting selection pressures by controlling what, and when, information is available to different observers (Cuthill et al. 2017; Postema et al. 2022; Kikuchi et al. 2023). These benefits may then be further facilitated by defensive behaviors and postures that can expose or conceal different components of an animal’s color (Stevens 2007; Drinkwater et al. 2022; Postema et al. 2022).

Batesian mimics also display seemingly-aposematic signals despite lacking secondary defenses, and take advantage of the avoidance behavior induced in predators by their defended models (Bates 1862). Selection on mimics is often assumed to favor ever more precise resemblance of the model as predators may learn to identify and exploit the remaining cues that distinguish palatable from defended prey (Kikuchi and Pfennig 2013). In nature, however, mimicry is often imperfect and mimics rarely (if ever) truly replicate the exact properties of their model’s visual signals (Sherratt 2002). Such imperfect mimicry has variously been linked to relaxed selection, physiological constraints, and sensory or perceptual limitations (Sherratt and Peet-Paré 2017). Consequently, as aposematic signals are variable, and mistakes potentially costly, predators generalize avoidance over a range of phenotypes that encompasses, but may also exceed, the likely trait space occupied by the defended model (Lindström et al. 1997; Briolat et al. 2019). Mimetic phenotypes can then evolve to be within this *cone of protection* where coloration is similar enough to the model to benefit from generalized avoidance behavior from predators (Kikuchi and Pfennig 2010a, 2010b, 2013).

Like aposematism, the evolution of mimicry will also be affected by a complex mix of interacting selection pressures. Despite similarities in ultimate appearance, however, the exact combination and magnitude of pressures on the mimic will likely differ from those of the model. For example, aposematically colored prey experience predation pressure from naïve, specialized, and nutritionally stressed predators (Barnett et al. 2007; Halpin et al. 2014). Toxic prey may survive interactions where predators test the honesty of signals through taste-reject behavior (Gamberale-Stille and Guilford 2004), but undefended species are expected to experience stronger negative outcomes from encounters with their predators (Wiklund and Järvi 1982). The difference in the risk posed by predators may then shift the balance of selection on mimics further toward crypsis (Speed and Ruxton 2010; Kraemer and Adams 2014). Previous mathematical modeling finds that by imposing a cost upon increased detectability in a mimic species, the resultant optimized phenotype is (i) imperfect and (ii) less detectable than the model (Speed and Ruxton 2010). This raises the question of how mimics may mitigate costs of detectability through morphological or behavioral adaptations which reduce predator encounter rates, while still maintaining effective mimicry.

One possibility is that mimics make use of multicomponent or multifunctional signals to incorporate camouflage into their displays. Where predators generalize avoidance over a range of traits, mimics may be able to deviate from perfect resemblance in ways which reduce detectability while retaining sufficient elements that assure inclusion or generalization into the *cone of protection* (Wang et al. 2017). This may include duller colors, hidden signals, or higher spatial frequency patterns which are less detectable when viewed from a distance (Tullberg et al. 2005; Stevens et al. 2007; Kikuchi and Pfennig 2013; Barnett and Cuthill 2014). Reduced detectability may reduce encounter rates without necessarily reducing the efficacy of mimicry. However, if interactions with predators are sufficiently reduced by greater crypsis, imperfect mimicry could persist even under high predation risk where predators frequently test the honesty of aposematic signals. Indeed, a similar adaptive function of imperfect mimicry may also apply more widely where selection for visual communication, species recognition, and/or mate choice conflicts with selection towards perfect mimicry (i.e. character displacement; Kikuchi and Pfennig 2013).

Poison frogs (Dendrobatidae) offer some of the most vivid examples of multicomponent and multifunctional colors, with patterns that have been concurrently selected for aposematism, camouflage, territory defense, and mate choice (Crothers and Cummings 2013, 2015). For example, salient signals have been co-opted for both aposematism and intraspecific communication (Maan and Cummings 2008, 2009; Crothers and Cummings 2013, 2015), and colors may act as both camouflage and salient signaling depending on the viewing conditions (i.e. the light environment (Rojas et al. 2014) or viewing distance (Barnett et al. 2018)) or in response to specific site selection behaviors (Willink et al. 2014a). Moreover, where multiple selection pressures are at play simultaneously, their impact on coloration can vary across the body depending on the visual systems and primary viewing angles of different observers (Siddiqi et al. 2004; Woolenberg et al. 2008; Willink et al. 2014b). As such, intraspecific communication may be primarily directed at conspecifics inhabiting the same viewing plane, whereas different defensive strategies may be targeted towards aerial and terrestrial predators (Siddiqi et al. 2004; Maan and Cummings 2008; Woolenberg et al. 2008; Rojas and Endler 2013).

Mimicry has also evolved multiple times among the poison frogs and their close relatives (Darst and Cummings 2006; Yeager et al. 2012). One such example is *Allobates zaparo* (Aromobatidae) a nontoxic, polytypic, Batesian mimic of 2 similarly colored and chemically defended poison frogs: *Ameerega bilinguis* and *Am. parvula* (Dendrobatidae) (Darst and Cummings 2006; Darst et al. 2006; Mebs et al. 2018). Both toxic species share a red dorsum, whereas *Am. bilinguis* also displays bright yellow spots and is moderately toxic, *Am. parvula* lacks spots and is the more toxic of the 2. *Allobates zaparo* mimics both species simultaneously with a red dorsum, and exhibits yellow limb spots to bear particular resemblance to *Am. bilinguis* when sympatric (Darst and Cummings 2006; Darst et al. 2006). Predators readily learn to avoid both *Am. parvula* and *Am. bilinguis,* as well as *Al. zaparo* once educated on an *Ameerega* spp. model, suggesting that the red dorsum and yellow spots are both recognized as aposematic signals (Darst and Cummings 2006; Darst et al. 2006). Poison frogs are, however, not immune from predation and even highly defended species are at risk from predators naïve to, or willing to ignore, aposematic signals (Toledo et al. 2007; Willink et al. 2014b). This raises the question of how the differing trade-offs experienced by a model and a mimic may affect how the balance between saliency and camouflage is expressed through color, morphology, and behavior.

Here, we used visual modeling and screen-based detection trials to examine how different components within the color patterns of *Am. bilinguis* and *Al. zaparo* affect detectability against the natural leaf litter background and from different viewing angles. Specifically, we asked whether *Am. bilinguis* and *Al. zaparo* differed in detectability, despite being grouped together by their predators, and whether viewing angle and defensive postures affect saliency. We predicted (1) that color patches primarily visible to predators (i.e. dorsal and spot colors viewed from above) would be less contrasting and more cryptic on the nontoxic mimic (*Al. zaparo*) than on the defended model *(Am. bilinguis*), (2) that viewing angle or body posture should affect relative detectability between model and mimic, by highlighting differences between model and mimic in regions less visible to aerial predators but visible to the frogs themselves (i.e. ventral colors viewed from the front versus hidden from the rear), and iii. that defensive behavior would decrease detectability, shifting the primary defense from signaling to camouflage.

## Methods

### Study system

**Fig. 1.**
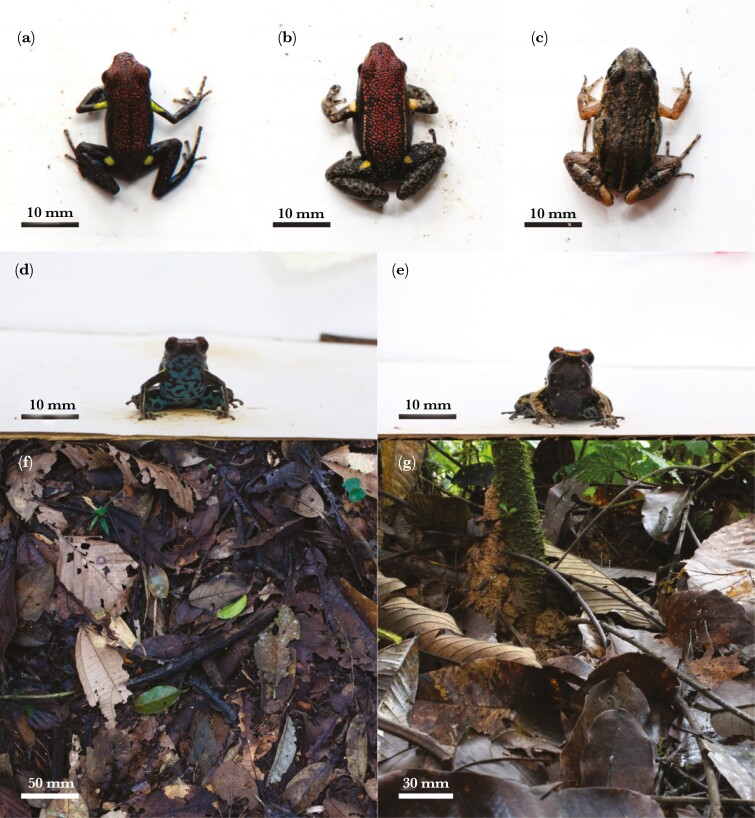
Study system. Top: dorsal view of the frogs photographed from the aerial perspective (a—poisonous *Am. bilinguis* (*model*), b—mimetic *Al. zaparo* (*mimic*), and c—cryptic *Ad. hylaedactyla* (*cryptic*)). Middle: anterior view of the frogs photographed from the terrestrial perspective (d—*Am. bilinguis* and E—*Al. zaparo*). Bottom: examples of the leaf litter background photographed from above (f—aerial) and from the side (g—terrestrial).

In May—July 2019, we measured the colors of 3 sympatric species of terrestrial frog and their natural forest floor habitat at the Iyarina Forest Reserve, Provincia de Napo, Ecuador (Fig. 1): the poisonous *Am. bilinguis* (*n* = 20, hereafter ‘*model*’ species), the nontoxic, Batesian mimic *Al. zaparo* (*n* = 20, hereafter ‘*mimic*’ species), and the nontoxic, cryptically colored, *Adenomera* c.f. *hylaedactyla* (Leptodactylidae, *n* = 20, hereafter ‘*cryptic*’ species). The study was conducted outside of the natural range of *Am. parvula,* and we included *Ad. hylaedactyla* as a nonaposematic and camouflaged leaf litter species against which we could compare the detectability of *Am. bilinguis* and *Al. zaparo.*

All 3 species have similar habitats (rainforest leaf litter), diets (myrmecophagy), and size classes (mean snout-vent length ± SD in our sample: *model* species 22.51 ± 1.63 mm, *mimic* species 26.92 ± 1.84 mm, and *cryptic* species 23.42 ± 2.69 mm) (Caldwell 1996; Darst et al. 2005). Defensive coloration has, therefore, likely evolved within the same visual environment and under the influence of a similar predator community that includes birds and reptiles (Toledo et al. 2007; Willink et al. 2014b). The *model* species has a red dorsum, a blue venter, and bright yellow front (axillary) and rear (inguinal) spots. The *mimic* species similarly has a red dorsum and yellow spots, but the venter appears dark grayish blue/black. The *cryptic* species is entirely mottled in dark gray and brown (Fig. 1A–E).

We searched for frogs during diurnal and nocturnal surveys of the leaf litter (Anderson et al. 2021; Barnett et al. 2023). Frogs were collected with the aid of a 50 mL plastic cup to avoid direct skin contact with the frogs. The *model* was handled with nonpowdered rubber gloves to avoid exposure to toxins.

The Iyarina Forest Reserve is a majority secondary rainforest, with a loose and interrupted canopy which allows daylight onto the forest floor. To measure coloration, we used spectrophotometry and calibrated photography of both the frogs and their environment. Spectrophotometry allowed for high resolution measurement of reflectance values from point sources (300–700 nm), whereas photography allowed us to capture the spatial structure of color and pattern (400–700 nm).

### Ethics

All work was conducted with permission from the Ministerio del Ambiente, Ecuador (permit: 014-2019-IC-FLO-DNB/MA). Experiments with animals were approved by the McMaster Animal Research Ethics Board (AREB#: 18-05-20) and experiments with human participants were approved by the McMaster Research Ethics Board (MREB#: 3781) at McMaster University, ON, Canada.

### Spectrophotometry

We recorded reflectance values from the *model* (*n* = 10) and *mimic* (*n* = 10) with an Ocean Optics Flame S-XR1-ES (200–1025 nm) spectrophotometer and DH-Mini Deuterium Tungsten Halogen (200–2500 nm) coaxial fibreoptic light source (Ocean Optics Inc.). We hand-held the probe at a 45° angle, 3 mm away from the sample and averaged across three 5 s scans for each measurement. For each frog, we took 3 measurements from the dorsum, 3 from the venter, and 2 from each of 2 front spots and 2 rear spots. To assess whether UV reflectance is an important component of the frogs’ coloration, we then used visual modeling to compare perceived contrast between observers with (UVS) and without (VIS) UV-sensitive vision in R package *Pavo* (Maia et al. 2019; Yeager and Barnett 2020). We found the influence of UV reflectance to be minimal and so we used the photographs to model visual contrast (Yeager and Barnett 2021; please see the Supplementary Material for details and reflectance spectra; Fig. S1).

### Photography

We photographed the frogs and their natural leaf litter backgrounds from 2 orientations: *aerial*, as viewed from above, and *terrestrial*, as viewed from the side. In the aerial photographs we included all 3 species to assess the detectability of the dorsal and spots colors. In the terrestrial photos we focused on the *model* and *mimic*, and excluded the *cryptic* species, as we were primarily interested in differences in ventral coloration within the mimicry system.

All photographs were taken under natural diffuse daylight conditions, with a Nikon D7200 DSLR camera and AF-S DX NIKKOR 35 mm lens (Nikon Corp. Japan). Each photo contained a ColorChecker Passport (X-Rite Inc., USA) to enable color calibration and scaling (Stevens et al. 2007; Troscianko and Stevens 2015).

We took the aerial frog photographs from a height of ~30 cm with each frog presented against a white background (*n* = 20 each *model*, *mimic*, and *cryptic* species). We first photographed a dorsal view of each species in its natural resting posture with their legs retracted, and then photographed the dorsal and ventral colors of the *model* and *mimic* species with their legs extended to reveal the spots (Fig. 1A–E). We took the aerial leaf litter photographs (*n* = 505) from a height of ~140 cm. We walked a nonlinear path through the rainforest and, at ~2 to 5 m intervals, captured patches of leaf litter (~50 cm × 70 cm) that were not occluded by plant growth (Fig. 1F; (Barnett et al. 2023)).

We took the terrestrial photographs of the *model* and *mimic* species (*n* = 20 each) in a white-walled photo booth (20 cm × 15 cm × 15 cm). The camera was placed 30 cm from the booth with the lens held parallel to the ground. We photographed each frog from 4 viewing orientations: from the front (anterior), from behind (posterior), and from each side (left lateral and right lateral). For 10 individuals of both species, we also photographed an anterior view of the frogs in a defensive crouched position that hid the ventral colors (crouched). We photographed the terrestrial leaf litter (*n* = 500) by placing the camera close to the ground, ~50 cm from piles of fallen leaves (~ 40 cm × 30 cm) that had naturally built up against tree roots and fallen timber—areas where frogs were frequently encountered (Fig. 1G).

### Visual modeling

To evaluate our first prediction that aerially visible coloration would be less conspicuous on the mimic than on the model, we computed perceived contrasts between the frogs’ components (e.g. spots against dorsum) and between each component and the background. These contrasts were obtained through visual modeling using the MICA Toolbox in ImageJ v1.53e (Schneider et al. 2012; Troscianko and Stevens 2015). We selected regions of interest (ROIs) from the aerial photographs that covered the dorsal color of all 3 species, then the front spot, rear spot, and ventral colors of the *model* and *mimic*, and a region of leaf litter of ~50 cm  × 70 cm. We then modeled bird (Eurasian blue tit*, Cyanistes caeruleus* (Hart et al. 2000)) and snake (coachwhip*, Masticophis flagellum* (Macedonia et al. 2009)) vision to evaluate contrast to aerial and terrestrial predators, respectively. We also modeled poison frog (strawberry poison frog, *Oophaga pumilio* (Siddiqi et al. 2004)) vision to approximate conspecific vision, and human vision (Smith and Pokorny 1975) to contextualize the results of our detection experiments.

Using the MICA Toolbox, we calculated chromatic (hue: ΔS) and achromatic (luminance: ΔL) contrast from the receptor-noise-limited visual discrimination model, in a manner akin to Just Noticeable Differences (JNDs; (Vorobyev and Osorio 1998; Troscianko and Stevens 2015); see the supplementary material). Colors with ΔS or ΔL values of <1 are unlikely to be discriminated under ideal viewing conditions, colors with values between 1 and 3 are closely matched, and values >3 indicate that colors can increasingly be differentiated (Walton and Stevens 2018).

We first measured the contrast found between the color patches (*Internal Contrast*) of each individual *model* and *mimic* by calculating ΔS and ΔL between the dorsum and spots (aerial view) and between the dorsum and venter (terrestrial view). We compared internal pattern contrast between the species with 2 pairs of linear models, using the function *lm* from base R v4.2.2 (R Core Team 2022). Each model included ΔS or ΔL (log-transformed if necessary to fit model assumptions) as the response variable, *species* as the fixed effect, and we checked model assumptions with the *check_model* function from R package *performance* (Lüdecke et al. 2021).

To assess detectability against the leaf litter (*External Contrast*), we paired each individual frog with a single randomly selected background image. For each frog, we then calculated ΔS and ΔL between the background ROI and each component of the frogs’ coloring. As with *Internal Contrast*, we analyzed differences in external contrast between the species with a series of 8 linear models from base R v4.2.0 and we checked model assumptions using R package *performance* (Lüdecke et al. 2021). Our response variables were: (1) dorsal ΔS, (2) dorsal ΔL, (3) front spot ΔS, (4) front spot ΔL, (5) rear spot ΔS, (6) rear spot ΔL, (7) ventral ΔS, and (8) ventral ΔL (log-transformed if necessary to fit model assumptions), and each model included *species* as the fixed effect. As our dorsal comparison included all 3 species we performed additional pairwise contrasts using R package *multcomp,* with *P* values corrected using the single-step method (Hothorn et al. 2008).

In our initial analysis of External Contrast, each background ROI averaged across ~3,500 cm^2^ of leaf litter. Consequently, it is possible that we are underestimating background heterogeneity and comparing frog color regions to averaged hue and luminance values that are not themselves present in the background. We therefore repeated the analysis using smaller, frog-sized (2.5 cm × 2.5 cm), background ROIs (*n* = 60). In this alternate analysis we compared each frog to each of the background ROIs, and included frog ID and background ID as random factors in the model (see the Supplementary Material).

### Detection experiments with human observers

To corroborate our visual modeling estimates of signal contrast, and to investigate how frog coloration influences detectability in natural scenes, we conducted 2 detection experiments with human participants: 1 from the aerial perspective and 1 from the terrestrial perspective. Both experiments were conducted online using participants recruited through the undergraduate research participation program at McMaster University. In each experiment, we presented frogs, scaled to their natural size with different color manipulations, against their natural backgrounds. Participants were tasked with clicking on the frogs as quickly as possible with the mouse. We then recorded the time taken for each correct identification (*response time*) as our metric of detectability.

It is important to note that there are significant differences in visual perception between humans and natural observers (e.g. predators) that have driven frog color evolution (snakes, Hauzman 2020; birds, Kelber 2019; as predators—Toledo et al. 2007). However, there remain important similarities in basal visual processing and human participants have repeated been demonstrated to replicate target detectability data from wild birds, especially where UV reflectance in minimal, such as in our study (Barnett et al. 2016, 2018, 2020; Xiao and Cuthill 2016; Kjernsmo et al. 2020; Yeager and Barnett 2021).

### Aerial detection

In the aerial detection experiment we further tested our first prediction by quantifying the detectability of the *model*, *mimic*, and *cryptic* species’ dorsal coloration against the leaf litter background, as well as the spot coloration of the *model* and *mimic*. To make our experimental stimuli, we cropped the whole frog and all 4 spots from our aerial photographs. We then created all frog-spot combinations by combining the frog bodies (ABI = *model*, AZA = *mimic*, and AHY = *cryptic*) with the spots (BS = *model* spots, ZS *= mimic* spots) and with no spots (NS). In total we created 9 treatments (Fig. 3A). To make the experimental backgrounds we cropped an area ~70 cm  × 50 cm of leaf litter from the aerial photographs (Fig. 1F). For each participant, we randomly selected 5 of the 20 frogs per species to be used as the basis for the stimuli. We then combined each frog stimulus with a randomly selected background image, with frog location and orientation selected at random from uniform distributions. Each of the 9 treatments was presented 5 times for a total of 45 unique trials for each of the 108 participants.

We analyzed log-transformed *response time* with a linear mixed effects model in R package *lme4* (Bates et al. 2015). We included *treatment* as the fixed effect, *participant id* and *frog* id as random intercepts, and checked model assumptions with R package *performance* (Lüdecke et al. 2021). We then used the R package *multcomp* to perform a series of pairwise contrasts, designed to test specific *a priori* hypotheses (Hothorn et al. 2008). First, to test whether the dorsal colors of each frog were cryptic or conspicuous we compared each species with the spots concealed (ABI.NS vs AZA.NS vs AHY.NS). Second, to assess whether the unveiling of spots increased saliency we compared the *model* and *mimic* with and without their spots (ABI.BS vs ABI.NS | AZA.ZS vs AZA.NS). Third, to see whether the spots of the *model* were more conspicuous than those of the *mimic*, we compared each species with the *model’s* spots to itself with the *mimic’s* spots (ABI.BS vs ABI.ZS | AZA.BS vs AZA.ZS | AHY.BS vs AHY.ZS). Finally, we compared the natural configuration of each species, with spots exposed (if present) to see whether the *mimic* would be less detectable than the *model* under natural conditions (ABI.BS vs AZA.ZS vs AHY.NS). We corrected p values for multiple testing using the single-step method (Hothorn et al. 2008).

### Terrestrial detection

We conducted a terrestrial view detection experiment to evaluate our second prediction that model and mimic would differ most greatly in their ventral coloration, and that viewing angle or posture could modulate the detectability relationship between model and mimic, as well as our third prediction that defensive behavior (i.e. crouched posture) would decrease detectability. To investigate how viewing angle influences the detectability of the *model* and *mimic* to terrestrial observers, we had human participants search for stimuli created from the horizontally photographed leaf litter and the frogs photographed from different horizontal orientations. This created 10 treatments: ABI (the *model*) and AZA (the *mimic*) photographed in front-facing “anterior” (A), back-facing “posterior” (P), right-facing lateral (R), left-facing lateral (L), and an anterior view of the frog in a defensive crouched posture (C) (Fig. 4A). We then cropped an area representing ~40 cm × 35 cm of leaf litter from the terrestrial photographs to form the experiment backgrounds (Fig. 1G). In each terrestrial photo we restricted frogs to appear only in the foreground region to prevent stimuli from appearing against the canopy or a background region where scaling would be mismatched. As in the aerial detection experiment, frogs and backgrounds were randomly selected and combined, and each treatment combination was presented 5 times, for a total of 50 unique trials for each of the 101 participants.

We analyzed log-transformed *response time* with a linear mixed effects model that included *treatment* as a fixed effect, and *participant ID* as well as *viewing angle* nested within *frog ID* as random intercepts, using the R package *lme4*. We checked model assumptions using the R package *performance*. We then conducted pairwise contrasts, of *a priori* interest, using R package *multcomp* and corrected *P* values with the single-step method. First, to test for intra-specific differences in detectability between different viewing angles, for each species, we compared the posterior view to the left-lateral (ABI.P vs ABI.L | AZA.P vs AZA.L), right-lateral (ABI.P vs ABI.R | AZA.P vs AZA.R), and anterior (ABI.P vs ABI.A | AZA.P vs AZA.A) viewpoints. Second, to assess the effect of the defensive crouched posture on detectability, for both species, we compared the crouched posture to the anterior (ABI.C vs ABI.A | AZA.C vs AZA.A) and posterior (ABI.C vs ABI.P | AZA.C vs AZA.P) views. Finally, to evaluate inter-specific signal saliency, we compared the *model to* the *mimic* in the anterior (ABI.A vs AZA.A), posterior (ABI.P vs AZA.P), and crouched (ABI.C vs AZA.C) conditions.

## Results

### Visual modeling

Here, we report the results from the avian visual model, but these findings are qualitatively equivalent to those from the 3 other visual systems modeled (see the Supplementary Material for the snake, poison frog, and human models).

### Visual modeling: internal contrast

**Fig. 2.**
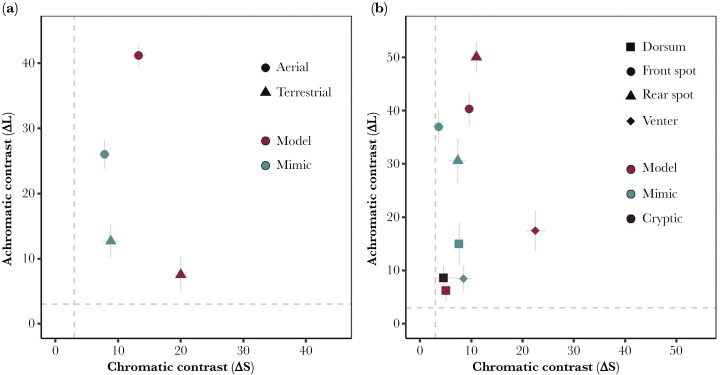
Visual modeling (avian visual model). a) Internal contrast. Chromatic (hue: ΔS) and achromatic (luminance: ΔL) contrast (means ± 95% CI from the raw data) found within the patterns of *Am. bilinguis* (*model*; red) and *Al. zaparo* (*mimic*; blue) when viewed from above (circles = aerial perspective (dorsum vs spots)) and from the side (triangles = terrestrial perspective (dorsum vs venter)). From above *Am. bilinguis* is more contrasting in both ΔS and ΔL. From the side, *Am. bilinguis* has greater ΔS but *Al. zaparo* has higher ΔL. b) External contrast. Chromatic (ΔS) and achromatic (ΔL) contrast (means ± 95% CI from the raw data), between the colors (circle = dorsum, triangle = front spot, square = rear spot, and diamond  = venter) of the frogs *Am. bilinguis* (*model*), *Al. zaparo* (*mimic*), and *Ad. hylaedactyla* (*cryptic*) and the leaf litter. The dorsal colors of each frog, and the venter of *Al. zaparo,* closely match the background. The venter of *Am. bilinguis,* and the spots of *Am. bilinguis* and *Al. zaparo* are more distinct from the background. On both plots the grey dotted lines represent the visual discrimination threshold equivalent to 3 JND. See the Supplementary Material for the results from the snake, poison frog, and human models.

When analyzing within-individual contrast, we found the internal signal of the *model* to generally be more contrasting than those of the *mimic* to an aerial observer. The *model’s* spot-dorsum contrast was higher for both chromatic ($F_{38}^{1}$ = 91.00, *P* < 0.001) and achromatic ($F_{38}^{1}$ = 115.90, *P* < 0.001) contrast. Whereas to a terrestrial observer, the 2 species were highly contrasting in different ways. When comparing contrast between the dorsum and venter, the *model* had stronger chromatic contrast ($F_{38}^{1}$ = 270.55, *P* < 0.001) but the *mimic* had stronger achromatic contrast ($F_{38}^{1}$ = 8.17, *P* = 0.007). All internal contrasts measured were well above the visual discrimination threshold (Fig. 2A).

### Visual modeling: external contrast

When analyzing external contrast, we found that the size of the background ROI did not qualitatively change the results, and so report results from the larger ROIs here (see the Supplementary Material for all other analyses).

When comparing the dorsal colors of the 3 species to the leaf litter background, we found a significant effect of species for both chromatic (Δ*S*: $F_{57}^{2}$ = 5.69, *P* = 0.006) and achromatic (Δ*L*: $F_{57}^{2}$ = 11.10, *P* < 0.001) contrast (Fig. 2B). Pairwise comparisons revealed that the red dorsal color of the mimic most strongly contrasted against the leaf litter, with significantly higher chromatic and achromatic contrast than the model (ΔS: *t* = −2.64, *P* = 0.028, Δ*L*: *t* = −4.56, *P* < 0.001) and cryptic species (ΔS: *t* = −3.14, *P* = 0.007, Δ*L*: *t* = −3.31, *P* = 0.005). However, there was no difference in chromatic (Δ*S*: *t* = 0.50, *P* = 0.873) or achromatic contrast (Δ*L*: *t* = 1.25, *P* = 0.431) between the model and cryptic species.

For all other components of coloration, the *model* was more contrasting against the leaf litter than the *mimic*. The *model’s* yellow front spots had stronger contrast than the *mimic’s* in chromatic ($F_{38}^{1}$ = 78.20, *P* < 0.001) but not achromatic ($F_{38}^{1}$ = 2.29, *P* = 0.138) contrast. The yellow rear spots of the *model* were more distinct in both chromatic ($F_{38}^{1}$ = 15.28, *P* < 0.001) and achromatic ($F_{38}^{1}$ = 47.02, *P* < 0.001) contrast. Similarly, the ventral color of the *model* had stronger chromatic and achromatic background contrast than that of the *mimic* (Δ*S*: $F_{38}^{1}$ = 142.55, *P* < 0.001 Δ*L*: $F_{38}^{1}$ = 18.45, *P* < 0.001).

### Detection 1: aerial observers

**Fig. 3.**
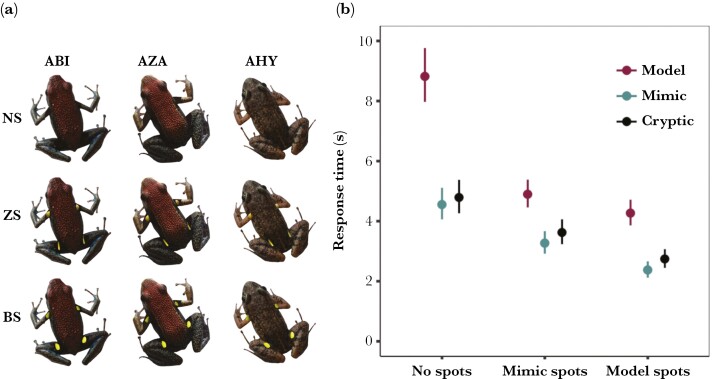
Aerial detection experiment (human participants). a) Example treatment designs (ABI = *Am. bilinguis* (*model*), AZA = *Al. zaparo* (*mimic*), AHY = *Ad. hylaedactyla* (*cryptic*) | NS = no spots, ZS = *Al. zaparo* (*mimic*) spots, BS = *Am. bilinguis* (*model*) spots). b) Response time (means ± 95% CI from the model) for each species (point order: Model—ABI , Mimic—AZA, Cryptic—AHY) and spot treatment (left to right: no spots—ABI.NS, AZA.NS, AHY.NS | mimic spots—ABI.ZS, AZA.ZS, AHY.ZS | model spots—ABI.BS, AZA.BS, AHY.BS). There was a significant effect of dorsal color and spot type, with the *model* having the most cryptic dorsum and most detectable spots.

In the aerial detection experiment, we found a significant effect of treatment on the time taken to find frogs of different color configurations (Table 1; Fig. 3). We, therefore, conducted pairwise contrasts to investigate: (1) how detectable the dorsal patterns of each species were when the spots were hidden, (2) how the presence of spots affected the detectability of *model* and *mimic*, (3) how the *model’s* spots differed from the *mimic’s* spots with fixed dorsal coloration, and (4) how the natural patterns (spots exposed, if present) of each species differed in detectability (Table 1; Fig. 3; Fig. S6).

**Table 1.** Results from the aerial detection experiment.

*Main effect of treatment*	*χ* ^2^ = 584.18, df = 8, *P* < 0.001
*Dorsal colors (spots hidden)*	
ABI (NS) vs AZA (NS)	*z* = 10.72, *P* < 0.001
ABI (NS) vs AHY (NS)	*z* = 9.83, *P* < 0.001
AZA (NS) vs AHY (NS)	*z* = -0.85, *P* = 0.974
*Spots visible vs spots hidden*	
ABI (BS) vs ABI (NS)	*z* = −14.12, *P* < 0.001
AZA (ZS) vs AZA (NS)	*z* = 6.91, *P* < 0.001
*ABI spots vs AZA spots*	
ABI (BS) vs ABI (ZS)	*z* = -2.88, *P* = 0.038
AZA (BS) vs AZA (ZS)	*z* = -6.48, *P* < 0.001
AHY (BS) vs AHY (ZS)	*z* = -5.91, *P* < 0.001
*Natural patterns* (spots exposed, if present)	
ABI (BS) vs AZA (ZS)	*z* = 4.57, *P* < 0.001
ABI (BS) vs AHY (NS)	*z* = -1.96, *P* = 0.351
AZA (ZS) vs AHY (NS)	*z* = -6.48, *P* < 0.001

*Note*: Treatment codes for species (ABI = *Am. bilinguis* (*model*), AZA = *Al. zaparo* (*mimic*), & AHY = *Ad. hylaedactyla* (*cryptic*)) and spot manipulations (NS = target with no spots, ZS = target with *Al. zaparo* spots, BS = target with *Am. bilinguis* spots).

First, we found that the *model’s* dorsal color was the least detectable, with detection time being significantly longer than for the dorsal colors of both *mimic* (ABI.NS > AZA.NS) and *cryptic* species (ABI.NS > AHY.NS). Yet, we found no difference in the time taken to detect the dorsal colors of the *mimic* and *cryptic* species (AZA.NS = AHY.NS).

Second, we found the spots to be particularly salient features. The addition of each species’ naturally occurring spots significantly decreased detection time for both *model* (ABI.NS > ABI.BS) and *mimic* (AZA.NS > AZA.ZS) compared with when spots were absent. Moreover, the *model’s* spots were more conspicuous and decreased detection times more than the *mimic’s* spots. All 3 species’ detection times were significantly shorter when their dorsal colors were combined with the *model’s* spots than with the *mimic’s* spots (ABI.BS < ABI.ZS | AZA.BS < AZA.ZS | AHY.BS < AHY.ZS).

Finally, when the dorsal and spot colors were presented in their natural configurations, the *mimic* was the most detectable species. We found that the *mimic* was detected significantly more quickly than both the *cryptic* (AZA.ZS < AHY.NS) and the *model* species (AZA.ZS < ABI.BS). However, even when its bright spots were exposed, there was no significant difference in detection time between the *model* and the *cryptic* species (ABI.BS = AHY.NS).

### Detection 2: terrestrial observers

**Fig. 4.**
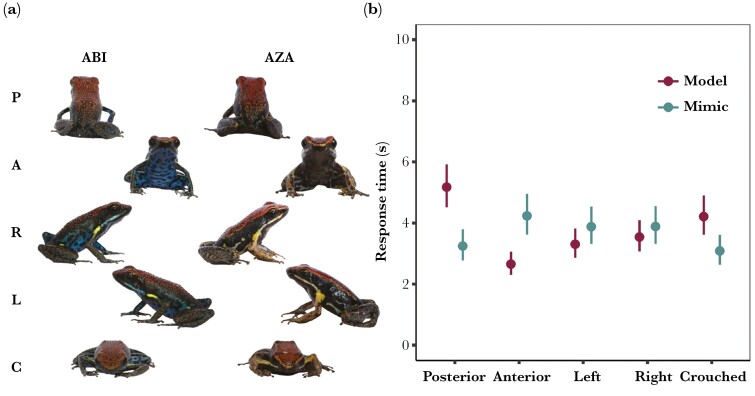
Terrestrial detection experiment (human participants). a) Example treatment designs (ABI = *Ameerega bilinguis* (*model*), AZA = *Allobates zaparo* (*mimic*) | P = posterior, A = anterior, R = right lateral, L = left lateral, C = crouched). b) Response time (means ± 95% CI from the model) for each combination of species (point order: Model—ABI , Mimic—AZA) and viewing angle (left to right: posterior—ABI.P, AZA.P | anterior—ABI.A, AZA.A | left lateral—ABI.L, AZA.L, right lateral—ABI.R, AZA.R | crouched—ABI.C, AZA.C). Viewing angle significantly affects detection time but the trends differ between species: the *model* is easiest to find when showing its venter and hardest to find when showing its dorsum, whereas the *mimic* is easiest to find when showing its dorsum and hardest to find when showing its venter.

In the terrestrial detection experiment, there was a significant effect of treatment on the time taken to find frogs facing in different directions (Table 2; Fig. 4). We, therefore, conducted pairwise comparisons to examine: (1) how viewing angle affected the detectability of the *model* and *mimic* relative to the dorsal pattern (posterior view), (2) how crouching behavior changed detectability relative to the ventral (anterior view) and dorsal (posterior view) colors, and (3) how the model and mimic differed in detectability when viewed from the front (anterior view), from the back (posterior view), and when in the defensive posture (crouched) (Table 2; Fig. 4; Fig. S7).

**Table 2.** Results from the terrestrial detection experiment.

*Main effect of treatment*	*χ* ^2^ = 93.85, df = 9,*P* < 0.001	
*Viewing orientation*		
	ABI	AZA
Back to front: (P) vs (A)	*z* = −9.23, *P* < 0.001	*z* = −3.707, *P* = 0.003
Back to left: (P) vs (L)	*z* = 6.16, *P* < 0.001	*z* = −2.51, *P* = 0.137
Back to right: (P) vs (R)	*z* = 5.215, *P* < 0.001	*z* = −2.52, *P* = 0.136
Lateral: (L) vs (R)	*z* = -0.96, *P* = 0.978	*z* = 0.018, *P* > 0.999
*Crouching behaviour*		
	ABI	AZA
Front to crouched: (A) vs (C)	*z* = −6.12, *P* < 0.001	*z* = 4.34, *P* < 0.001
Back to crouched: (P) vs (C)	*z* = 2.68, *P* = 0.091	*z* = 0.69, *P* = 0.998
*Comparing between model and mimic*		
Anterior: ABI (A) vs AZA (A)	*z* = 5.984, *P* < 0.001	
Posterior: ABI (P) vs AZA (P)	*z* = -5.905, *P* < 0.001	
Crouched: ABI (C) vs AZA (C)	*z* = 3.755, *P* = 0.002	

*Note*: Treatment codes for species (ABI = *Am. bilinguis* (*model*) and AZA = *Al. zaparo* (*mimic*)) and viewing angle (P = posterior, A = anterior, L = left lateral, R = right lateral, and C = crouching posture).

We found that the model was least detectable when observed from behind, and detection time was significantly longer than the front (ABI.P > ABI.A), left lateral (ABI.P > ABI.L), and right lateral (ABI.P > ABI.R) viewing angles. Conversely, we found that the mimic was most detectable when viewed from behind, with detection time being significantly shorter than the front (AZA.P < AZA.A), right (AZA.P < AZA.R), and left lateral (AZA.P < AZA.L) viewing angles. For both species, there was no significant difference between the left and right lateral views (ABI.L = ABI.R | AZA.L = AZA.R).

Crouching hides the ventral colors but exposes the dorsal colors, and we found that such behavior significantly altered detectability for both model and mimic. Compared with the ventral colors (anterior view), crouching significantly decreased detectability for the model (ABI.A > ABI.C) but increased detectability for the mimic (AZA.A < AZA.C). We also found that the crouched and rear views were equally detectable for both model and mimic (ABI.C = ABI.P | AZA.C = AZA.P).

Finally, when comparing between the 2 species, we found that relative detectability differed depending on viewing angle. When their ventral colors were exposed towards the observer, the *model* was more detectable than the *mimic* (ABI.A < AZA.A). However, when viewed from behind or when crouched down, where the dorsal colors were emphasized, the *mimic* was more detectable than the *model* (ABI.P > AZA.P | ABI.C > AZA.C).

## Discussion

Taken together our results reveal a balance between aposematic signaling and camouflage for both the chemically defended *model* and the nontoxic Batesian *mimic.* In accordance with our first prediction, the spot coloration of the *model* was more salient than that of the *mimic*, as was the internal signal contrast. Opposing our first prediction, however, we find the dorsal signal and natural (spot and dorsal) phenotype of the *mimic* to be more detectable than that of the *model*. In support of our second prediction, we find that the ventral coloration of the *model* and *mimic* are very different in their salience, with the *model* having a high-contrast venter and the *mimic’s* being much less salient, and that viewing angle and body posture modulate the detectability relationship between the 2 species. We find support for our third prediction in that concealment of the spots greatly reduced detectability in *model* and *mimic*, as well as that a defensive crouched posture, concealing the salient ventral coloration, decreased detectability in the *model*. However, we found the opposite of this effect for the mimic. These results suggest that for both species color may facilitate multiple defensive strategies, and that these functions may differ depending on viewing angle and the frogs’ behavior. However, despite being similar in appearance, and being confused for one another by avian predators (Darst and Cummings 2006; Darst et al. 2006), this interaction between defensive signaling and camouflage appears to differ between the 2 species. As a result, our data suggest that imperfect mimicry does affect the observers’ likelihood of detecting both species.

When viewed from above by aerial observers, the *model* and *mimic* both display a similar pattern with a red dorsum and yellow spots. We found that the dorsal colors of the 2 species were cryptic, having low contrast against the leaf litter and being no more detectable than the dorsal color of the *cryptic* species. Conversely, the yellow spots of the *model* and *mimic* were highly salient and substantially increased detectability. Frog behavior, therefore, also plays an important role in signal perception, as *model* and *mimic* were both more cryptic when the spots were hidden. From these results we suggest that camouflage is the initial defense utilized by both species, with the bright yellow spots acting as a salient warning which may be facultatively concealed or revealed through changes in posture. Future work is needed to discern whether exposure of spots at close range or in motion in these frogs elicits stronger predator avoidance.

It is important to note that, despite displaying a similar red dorsum, the third member of the mimicry system *Am. parvula* entirely lacks spots. Yet, birds will still learn to avoid *Am. bilinguis* and *Al. zaparo* (including forms with and without spots) from experience with *Am. parvula* (Darst and Cummings 2006; Darst et al. 2006). These results suggest that the red dorsum is aposematic, and so, when combined with our findings, likely acts as a multifunctional trait which initially provides camouflage and then conveys important information to predators postdetection (Honma et al. 2015; Postema et al. 2022). Additionally, the co-model *Am. parvula* is of higher toxicity than our study’s *model, Am. bilinguis* (Darst and Cummings 2006). This difference in toxicity may shift the signaling balance for *Am. bilinguis* further towards crypsis compared with its ‘stronger’ co-model, evolving a darker dorsum but incorporating bright spots that can be facultatively concealed or revealed to modulate detectability. Future testing is needed to determine whether *Am. parvula’s* dorsum is of higher salience than that of *Am. bilinguis*, and whether this is influenced by differences in toxicity between the 2 aposematic species.

Despite similarities in coloration, there were significant differences in internal signal contrast saliency when comparing *model* and *mimic*. As was expected, the *model’s* spots were more salient than those of the *mimic*. However, the *model’s* more cryptic dorsum meant that, contrary to our predictions, the *mimic* was more easily detected both when spots were hidden and exposed. Postdetection, the *model’s* brighter spots and darker dorsum does mean that its internal pattern contrast is stronger than the *mimic’s*. This may lend the *model* a more intense and effective signal at close range (Prudic et al. 2007; Halpin et al. 2020) or when the spots are suddenly revealed (Drinkwater et al. 2022), such as during predator handling, without necessarily increasing the distance at which they are initially detected Again, further work is however needed to understand how frog behavior may change during direct interactions with predators.

We found mixed support for our hypothesis when frogs were viewed at different angles from the terrestrial perspective; the likely view of not only predators such as snakes but also conspecific and heterospecific frogs. Owing to its bright blue ventral coloration and cryptic dorsum, the *model* was most detectable from the front. In contrast, due to its dark venter and comparatively bright dorsum, the *mimic* was most detectable when viewed from the rear. Further, body posture once again played an important role in signal saliency. A defensive crouched posture hides the ventral colors, exposes the dorsal coloration, and reduces the profile of the frog. The *model* may adaptively conceal its conspicuous venter, as its detectability is decreased in the defensive crouch position compared with a frontal view. Opposingly, the *mimic* species increases in detectability from the front when crouched, presumably due to its cryptic ventral coloration being concealed in favor of its relatively brighter dorsum. These results suggest that imperfect mimicry can lead to circumstantial factors such as observer viewing angle and prey body posture differentially influencing the detectability of a model versus a mimic.

Mimicry is frequently imperfect in many different taxa such as insects, snakes, amphibians, and fish, among others, and morphological differences between models and mimics may arise as a result of factors including limitations in predator perception, developmental constraints, or relaxed selection (Kikuchi and Pfennig 2013; Sherratt and Peet-Paré 2017). Due to costs associated with salient signaling, mimics are largely assumed to have less conspicuous colors that their models (Stevens 2007; Kikuchi and Pfennig 2013). Curiously, however, when viewed from above or behind, we find the opposite, with the *mimic* being the more detectable species. This enhanced detectability of the *mimic* may owe to its brighter dorsum than that of the *model*, a signal discrepancy also observed in mimetic salamanders (Kraemer and Adams 2014). A more detectable mimic should be predicted to face relatively higher rates of predation detection than its model. This predicted increase in predation may not be realized, however, if predators in a specific habitat do not routinely test the honesty of aposematic signaling; i.e. sample-reject predation is infrequent (Gamberale-Stille and Guilford 2004; Skelhorn and Rowe 2006a, b).

As predicted, the ventral colors provided the most salient difference between *model* and *mimic*, with the *model’s* being highly detectable whereas the *mimic’s* was cryptic. Conspicuous ventral colors are common in chemically defended amphibians and may act as an aposematic signal primarily visible during close range interactions with predators (Loeffler-Henry et al. 2023). The presence of similar bright blue ventral colors in congeners of the *model* species (*Ameerega* spp.) outside of this mimicry system suggests this is an ancestral trait which is poorly matched by the *mimic* (Serrano-Rojas et al. 2017), rather than *chase-away selection* driving the evolution of novel elements in the *model’s* aposematic signal to reduce mimetic resemblance (Kikuchi and Pfennig 2013).

We should also recognize that additional functions of color, not directed at predators, may affect mimetic fidelity. The co-option of aposematic signals for important social or sexual functions has been well characterized (Rojas 2017, 2018), and brighter, more saturated colors are frequently favored during mate choice and territory defense in poison frogs (Maan and Cummings 2009; Crothers and Cummings 2015). We therefore cannot dismiss the possibility that social or sexual communication affects signal detectability differently in the *model* versus *mimic* species in our study. For example, dorsal brightness in the *mimic* may be exaggerated by selection for intraspecific signaling, perhaps facilitated by mimicry yet constrained by camouflage. Whereas, in the *model*, conspicuous ventral colors could signal to conspecific or heterospecific frogs, while being hidden from predators. Indeed, Anderson et al. (2021) found that the *model* and *mimic* will both attend to territory intruders of either species, and future research is needed to understand how intra- and inter- specific interactions/recognition may be mediated by species-specific visual cues such as ventral coloration.

Overall, we found that the colors of the *model* and *mimic* include multiple discrete components that fulfill cryptic and aposematic functions depending on context and behavior. In contrast to our predictions we find that a Batesian mimic may exhibit a more detectable phenotype than its model, suggesting that additional factors beyond crypsis may be at play. These results invite future testing for species recognition, mate choice, or territory defense functions. Our study contributes to a growing interest in how ecological pressures aside from predator avoidance can affect the evolution of aposematic coloration (Kikuchi et al. 2021; Postema et al. 2022; Kojima et al. 2024). We suggest that imperfect mimicry can evolve due to these pressures differing (1) between model and mimic, and (2). between different pattern components. To fully understand the evolution and ecological dynamics of imperfect mimicry, therefore, it is vital to examine color evolution within a multifaceted framework where multiple signaling functions and the appropriate range of behavioral, perceptual, and environmental contexts are considered.

## Supplementary Material

arae077_suppl_Supplementary_Material

## Data Availability

Analyses reported in this article can be reproduced using the data provided by McEwen et al (2023).
