# Supplementary material for: Detectability of a poison frog and its Batesian mimic depends on body posture and viewing angle
Source: Behav Ecol. 2024 Oct 4;35(6):arae077. doi: 10.1093/beheco/arae077 (PMC11520748; doi:10.1093/beheco/arae077)
Supplement: arae077_suppl_Supplementary_Material [file arae077_suppl_supplementary_material.docx]

Supplementary Material for:

**Detectability of a poison frog and its Batesian mimic depends on body posture and viewing angle**

**AUTHORS**

Brendan L. McEwen ^a^, Justin Yeager ^b^, Isaac Kinley ^a,c^, Hannah M. Anderson ^a^, & James B. Barnett, ^a, d^

**ADDRESS**

^a^ Department of Psychology, Neuroscience, & Behaviour, McMaster University, Hamilton, ON, Canada

^b^ Grupo de Investigación en Biodiversidad, Medio Ambiente y Salud (BIOMAS), Facultad de Ingenierías y Ciencas Aplicadas, Universidad de Las Américas, Quito, Ecuador

^c^ Rotman Research Institute at Baycrest, Toronto, ON, Canada

^d^ School of Natural Sciences, Trinity College Dublin, Dublin, Ireland

Correspondence to:

Brendan L. McEwen. Email: mceweb1@mcmaster.ca

James B. Barnett. E-mail: jbarnett@tcd.ie

**KEYWORDS**

Aposematism, Batesian mimicry, imperfect mimicry, detectability, poison frogs, visual ecology.

This supplementary file includes:

1. Spectrophotometry
   - Supplementary spectrophotometry methods
   - Figure S1 – reflectance curves and UVS vs VIS models
2. Visual Modelling
   - Supplementary visual modelling methods
3. Results - snake visual model
   - Table S1 – internal contrast statistics
   - Table S2 – external contrast statistics
   - Figure S2 – A: internal pattern contrast, B: external pattern contrast
4. Results – poison frog visual model
   - Table S3 – internal contrast statistics
   - Table S4 – external contrast statistics
   - Figure S3 – A: internal pattern contrast, B: external pattern contrast
5. Results - human visual model
   - Table S5 – internal contrast statistics
   - Table S6 – external contrast statistics
   - Figure S4 – A: internal pattern contrast, B: external pattern contrast
6. Results – re-analyzing background contrast using small leaf litter ROIs
   - Figure S5 – A: original bluetit external contrasts, B: alternate bluetit external contrasts
   - Table S7 – Alternate bluetit external contrast statistics
   - Figure S6 – A: original coachwhip external contrasts, B: alternate coachwhip external contrasts
   - Table S8: Alternate coachwhip external contrast statistics
   - Figure S7 – A: Original poison frog external contrasts, B: alternate poison frog external contrasts
   - Table S9: Alternate poison frog external contrast statistics
   - Figure S8 – A: Original human external contrasts, B: alternate human external contrasts
   - Table S10: Alternate human external contrast statistics
7. References
   - Supplementary references
8. **Spectrophotometry**

We used R package *Pavo* (Maia et al. 2019) to create two visual models based on the tetrachromatic UV-sensitive visual system of the Eurasian blue tit (*Cyanistes caeruleus*; Hart et al. 2000). The first model was UV-sensitive (UVS: 300–700 nm) and included all four single cones (λ_max_ UVS = 372, SWS = 448, MWS = 502, LWS = 563), the one double cone (λ_max_ = 563 nm), ocular media transmission data, natural (D65) daylight irradiance, Weber fractions of 0.05, and cone ratios of 1:2:2:3 (Hart et al. 2000; Maia et al. 2019). The second model (VIS: 400–700 nm) was not sensitive to UV reflectance and was identical except for the exclusion of the UVS cone.

We then used our two visual models to calculate chromatic (hue: ΔS) and achromatic (luminance: ΔL) contrast between the two frog species for each color region (dorsum, venter, front (axillary) spots, and rear (inguinal) spots). Chromatic and achromatic contrast were calculated using the Receptor Noise Limited Model in an equivalent manner to Just Noticeable Differences (JNDs) using the *bootcoldist* function from R package *Pavo* (Vorobyev & Osorio 1998; Hart et al. 2000; Maia et al. 2019). Chromatic contrast was calculated from the single cones, whereas achromatic contrast was calculated from the double cone. Higher values indicate that two colors are more likely to be differentiated, with 1 being the theoretical absolute visual discrimination threshold and values below 3 being difficult to discern under natural conditions (Vorobyev & Osorio 1998).

Our spectrophotometry revealed some evidence for UV reflectance (300–400 nm) from the spots and venter of *Am. bilinguis* (Figure S1). However, as the contribution of UV to total spot reflectance was minimal, and previous work suggests that UV reflectance does not significantly affect spot contrast, the presence of UV is unlikely to appreciably contribute to detectability when frogs are viewed from above or behind (Yeager & Barnett 2020; 2021). It did appear that UV reflectance could have a more significant effect on ventral color contrast. Yet, as *Am. bilinguis* was more reflective than *Al. zaparo* across the whole visual spectrum, removing UV would not change the direction of any differential detectability. As such, and as previous work suggests that the background does not reflect UV (Barnett et al. 2023), we used the photographs to model visual contrast. Nonetheless, we do caution that our analyses may underestimate the detectability of the ventral colors of *Am. bilinguis* to UV-sensitive observers.

**
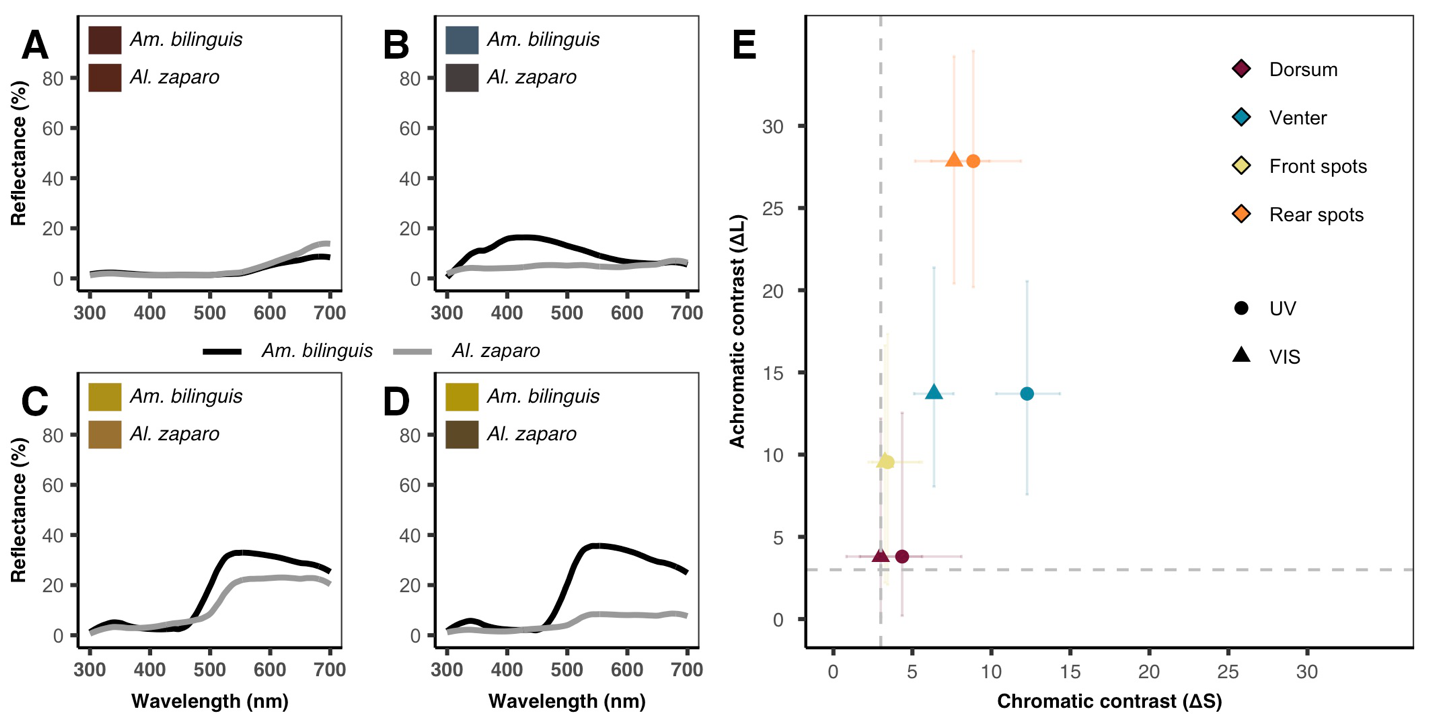
Figure S1. Spectrophotometry results**. A-D: Comparing mean spectral reflectance curves (300–700 nm) between *Am. bilinguis* (*model;* black) and *Al. zaparo* (*mimic;* grey), for the dorsum (A), venter (B), front (axillary) spots (C), and rear (inguinal) spots (D). Inset squares show reflectance curves converted into human visible colors. E: Comparing chromatic (ΔS) and achromatic (ΔL) contrast between the colors (dorsal, ventral, front spots, and rear spots) of *Am. bilinguis* and *Al. zaparo* as reported from UVS and VIS visual models (means ± 95% CI from the raw data). Shape indicates the visual model (circle = UV-sensitive; triangle = VIS-sensitive), color represents the body region (red = dorsal; blue = ventral; yellow = front (axillary) spots; orange = rear (inguinal) spots), and the grey dotted lines denote the estimated visual discrimination threshold equivalent to 3 JNDs.

1. **Visual Modelling**

When analyzing the photographs, we modelled the visual systems of a bird (Eurasian blue tit, *Cyanistes caeruleus*; λ_max_: UVS = 372, SWS = 448, MWS = 502, LWS = 563, Double = 563 nm; Hart et al. 2000) and a snake (coachwhip, *Masticophis flagellum*; λ_max_: UVS = 362, SWS = 458, LWS = 561 nm; Macedonia et al. 2009) to evaluate chromatic and achromatic contrast as viewed by aerial and terrestrial predators, respectively. We also modelled the visual system of a poison frog (*Oophaga pumilio*; λ_max_: SWS = 466, MWS = 489, LWS = 561 nm; Siddiqi et al. 2004)) to approximate conspecific vision, and human vision (λ_max_: SWS = 420, MWS = 489, LWS = 564 nm; Smith & Pokorny 1975) to contextualize the results of our detection experiments.

Chromatic contrast was calculated from the single cones and achromatic contrast was calculated using the double cone in the bird model, the LWS cone in the snake and poison frog models, and as the mean of the MWS and LWS cones in the human model. As UV reflectance was minimal, we excluded the UVS cone from the blue tit model. Weber Fractions, approximations of visual receptor noise, were set at 0.05 (Vorobyev & Osorio 1998; Siddiqi et al. 2004; Maan & Cummings 2012).

1. **Results - snake visual model**

**Table S1.** Results from the visual modelling of internal contrast.

|  | Chromatic Contrast (ΔS) | Achromatic Contrast (ΔL) |
| --- | --- | --- |
| Aerial view:  ABI vs AZA | $F_{38}^{1}$= 104.53, p < 0.001 | $F_{38}^{1}$= 135.75 p < 0.001 |
| Terrestrial view:  ABI vs AZA | $F_{38}^{1}$= 158.71, p < 0.001 | $F_{38}^{1}$= 2.76, p = 0.105 |

Note: Treatment codes for species (ABI = *Am. bilinguis*, AZA = *Al. zaparo*, & AHY = *Ad. hylaedactyla*). Viewing angle: aerial view = dorsum vs spots, terrestrial view = dorsum vs venter.

**Table S2.** Results from the visual modelling of external contrast (frog colors vs the whole leaf litter background).

|  | Chromatic Contrast (ΔS) | Achromatic Contrast (ΔL) |
| --- | --- | --- |
| Dorsal | $F_{57}^{2}$ = 1.61, p = 0.207 | $F_{57}^{2}$ = 8.79, p < 0.001 |
| ABI vs AZA | t = -1.53, p = 0.284 | t = -4.14, p < 0.001 |
| ABI vs AHY | t = 1.58, p = 0.261 | t = -1.50, p = 0.299 |
| AZA vs AHY | t = 0.052, p = 0.999 | t = 2.64, p = 0.028 |
| Front Spot  ABI vs AZA | $F_{38}^{1}$ = 37.08, p < 0.001 | $F_{38}^{1}$ = 2.42, p = 0.128 |
| Rear Spot  ABI vs AZA | $F_{38}^{1}$ = 19.83, p < 0.001 | $F_{38}^{1}$ = 48.37, p < 0.001 |
| Venter  ABI vs AZA | $F_{38}^{1}$ = 54.90, p < 0.001 | $F_{38}^{1}$ = 30.92, p < 0.001 |

Note: Treatment codes for species (ABI = *Am. bilinguis*, AZA = *Al. zaparo*, & AHY = *Ad. hylaedactyla*).

**
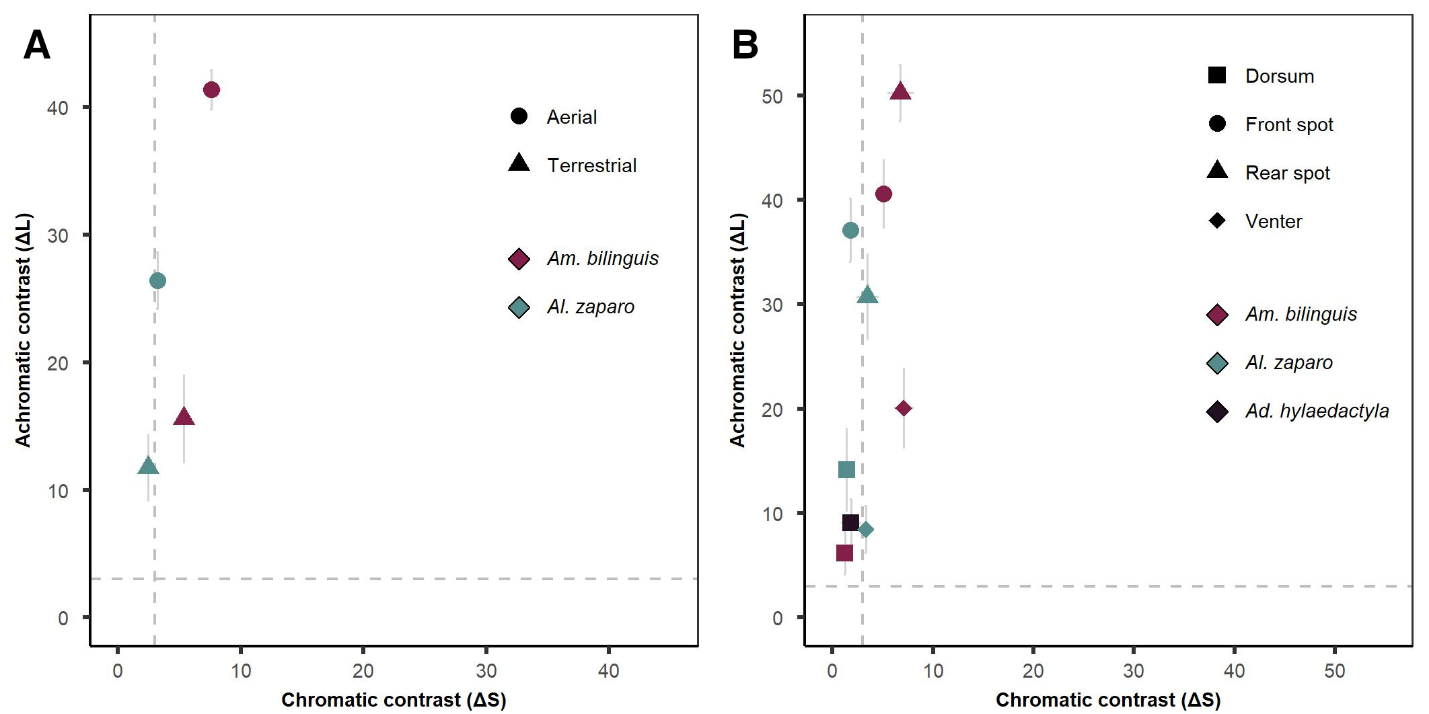
Figure S2: Visual modelling** (snake visual model). A. Internal contrast. Chromatic (ΔS) and achromatic (ΔL) contrast (means ± 95% CI from the raw data) found within the patterns of *Am. bilinguis* (red) and *Al. zaparo* (blue) when viewed from the aerial perspective (circles = dorsum vs spots) and from the terrestrial perspective (triangles = dorsum vs venter). B. External contrast. Chromatic (ΔS) and achromatic (ΔL) contrast (means ± 95% CI from the raw data), between the colors (square = dorsum, circle = front spot, triangle = rear spot, & diamond = venter) of the frogs (red = *Am. bilinguis*, blue = *Al. zaparo*, & black = Ad*. hylaedactyla*) and the whole leaf litter background. The grey dotted lines represent the visual discrimination threshold equivalent to 3 JND.

1. **Results – poison frog visual model**

**Table S3.** Results from the visual modelling of internal contrast.

|  | Chromatic Contrast (ΔS) | Achromatic Contrast (ΔL) |
| --- | --- | --- |
| Aerial view:  ABI vs AZA | $F_{38}^{1}$= 50.96, p < 0.001 | $F_{38}^{1}$= 111.96 p < 0.001 |
| Terrestrial view:  ABI vs AZA | $F_{38}^{1}$= 202.88 p < 0.001 | $F_{38}^{1}$= 1.04, p = 0.315 |

Note: Treatment codes for species (ABI = *Am. bilinguis*, AZA = *Al. zaparo*, & AHY = *Ad. hylaedactyla*). Viewing angle: aerial view = dorsum vs spots, terrestrial view = dorsum vs venter.

**Table S4.** Results from the visual modelling of external contrast (frog colors vs the whole leaf litter background).

|  | Chromatic Contrast (ΔS) | Achromatic Contrast (ΔL) |
| --- | --- | --- |
| Dorsal | $F_{57}^{2}$ = 2.46, p = 0.095 | $F_{57}^{2}$ = 9.78, p < 0.001 |
| ABI vs AZA | t = -2.20, p = 0.080 | t = -4.33, p < 0.001 |
| ABI vs AHY | t = 1.34, p = 0.381 | t = -1.39, p = 0.353 |
| AZA vs AHY | t = -0.87, p = 0.665 | t = 2.94, p = 0.013 |
| Front Spot  ABI vs AZA | $F_{38}^{1}$ = 22.93, p < 0.001 | $F_{38}^{1}$ = 2.42, p = 0.128 |
| Rear Spot  ABI vs AZA | $F_{38}^{1}$ = 8.45, p = 0.006 | $F_{38}^{1}$ = 48.02, p < 0.001 |
| Venter  ABI vs AZA | $F_{38}^{1}$ = 104.29, p < 0.001 | $F_{38}^{1}$ = 23.80, p < 0.001 |

Note: Treatment codes for species (ABI = *Am. bilinguis*, AZA = *Al. zaparo*, & AHY = *Ad. hylaedactyla*).


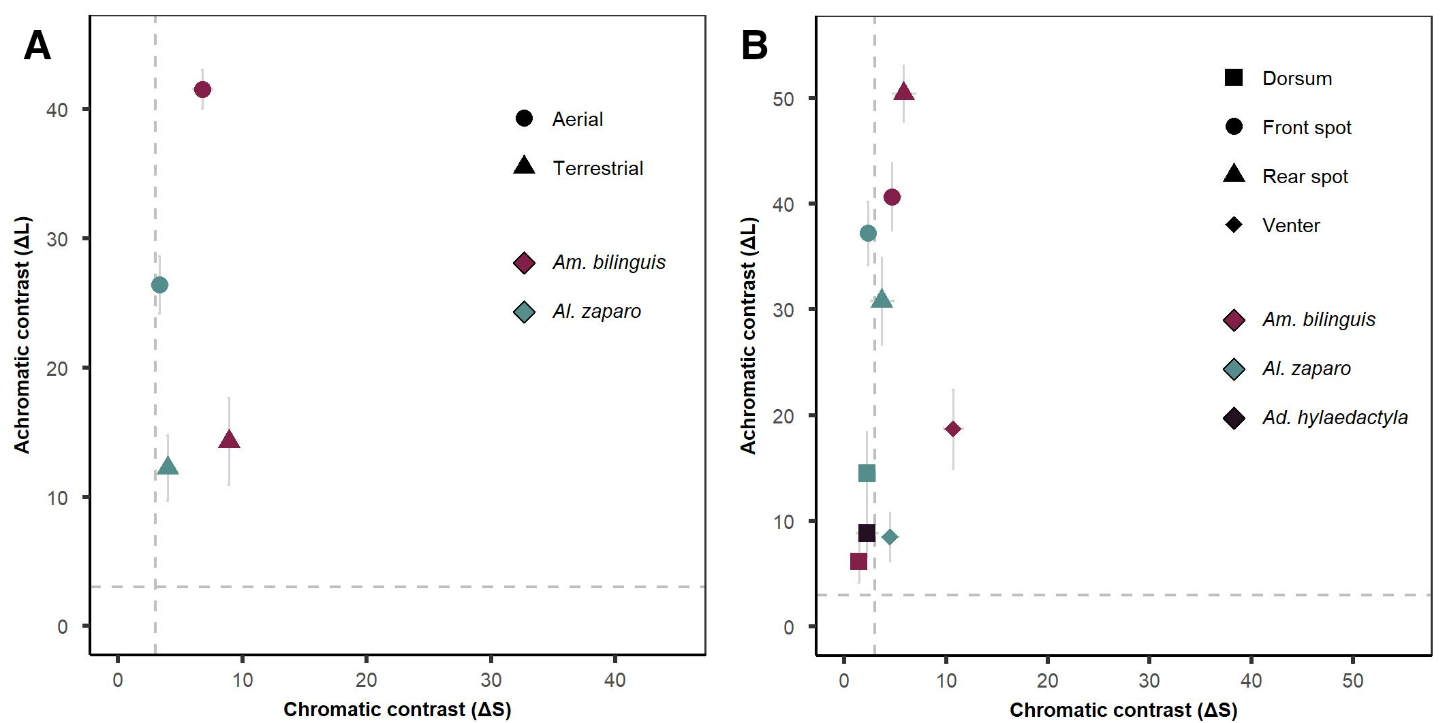
**Figure S3: Visual modelling** (poison frog visual model). A. Internal contrast. Chromatic (ΔS) and achromatic (ΔL) contrast (means ± 95% CI from the raw data) found within the patterns of *Am. bilinguis* (red) and *Al. zaparo* (blue) when viewed from the aerial perspective (circles = dorsum vs spots) and from the terrestrial perspective (triangles = dorsum vs venter). B. External contrast. Chromatic (ΔS) and achromatic (ΔL) contrast (means ± 95% CI from the raw data), between the colors (square = dorsum, circle = front spot, triangle = rear spot, & diamond = venter) of the frogs (red = *Am. bilinguis*, blue = *Al. zaparo*, & black = Ad*. hylaedactyla*) and the leaf litter background. The grey dotted lines represent the visual discrimination threshold equivalent to 3 JND.

1. **Results - human visual model**

**Table S5.** Results from the visual modelling of internal contrast.

|  | Chromatic Contrast (ΔS) | Achromatic Contrast (ΔL) |
| --- | --- | --- |
| Aerial view:  ABI vs AZA | $F_{38}^{1}$= 68.19, p < 0.001 | $F_{38}^{1}$= 121.56, p < 0.001 |
| Terrestrial view:  ABI vs AZA | $F_{38}^{1}$= 134.11, p < 0.001 | $F_{38}^{1}$= 0.45, p = 0.506 |

Note: Treatment codes for species (ABI = *Am. bilinguis*, AZA = *Al. zaparo*, & AHY = *Ad. hylaedactyla*). Viewing angle: aerial view = dorsum vs spots, terrestrial view = dorsum vs venter.

**Table S6.** Results from the visual modelling of external contrast (frog colors vs the whole leaf litter background).

|  | Chromatic Contrast (ΔS) | Achromatic Contrast (ΔL) |
| --- | --- | --- |
| Dorsal | $F_{57}^{2}$ = 3.36, p = 0.042 | $F_{57}^{2}$ = 9.83, p < 0.001 |
| ABI vs AZA | t = -1.24, p = 0.433 | t = -4.33, p < 0.001 |
| ABI vs AHY | t = -1.35, p = 0.375 | t = -1.36, p = 0.370 |
| AZA vs AHY | t = -2.59, p = 0.032 | t = 2.98, p = 0.012 |
| Front Spot  ABI vs AZA | $F_{38}^{1}$ = 43.86, p < 0.001 | $F_{38}^{1}$ = 3.91, p = 0.055 |
| Rear Spot  ABI vs AZA | $F_{38}^{1}$ = 5.70, p = 0.022 | $F_{38}^{1}$ = 49.80, p < 0.001 |
| Venter  ABI vs AZA | $F_{38}^{1}$ = 99.72, p < 0.001 | $F_{38}^{1}$ = 16.66, p < 0.001 |

Note: Treatment codes for species (ABI = *Am. bilinguis*, AZA = *Al. zaparo*, & AHY = *Ad. hylaedactyla*).


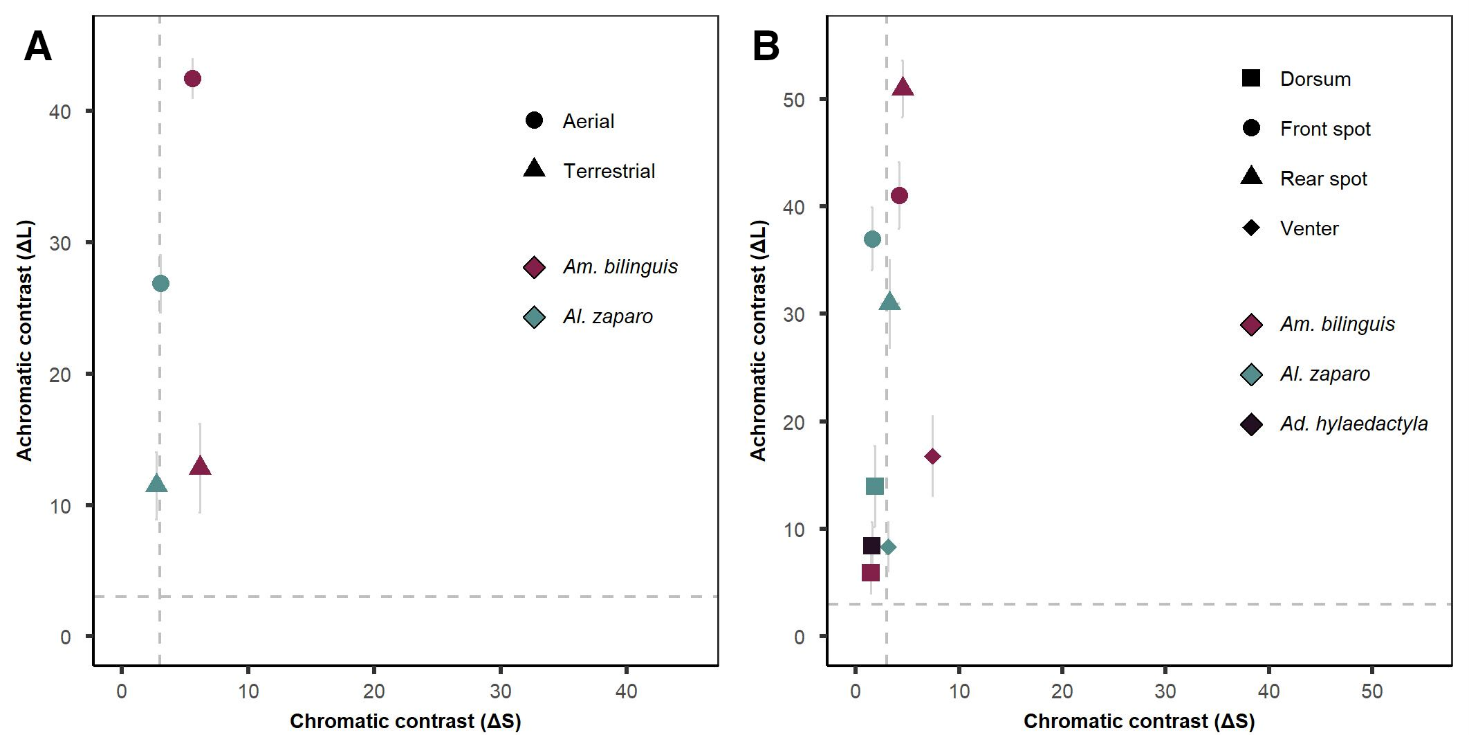
**Figure S4: Visual modelling** (human visual model). A. Internal contrast. Chromatic (ΔS) and achromatic (ΔL) contrast (means ± 95% CI from the raw data) found within the patterns of *Am. bilinguis* (red) and *Al. zaparo* (blue) when viewed from the aerial perspective (circles = dorsum vs spots) and from the terrestrial perspective (triangles = dorsum vs venter). B. External contrast. Chromatic (ΔS) and achromatic (ΔL) contrast (means ± 95% CI from the raw data), between the colors (square = dorsum, circle = front spot, triangle = rear spot, & diamond = venter) of the frogs (red = *Am. bilinguis*, blue = *Al. zaparo*, & black = Ad*. hylaedactyla*) and the leaf litter background. The grey dotted lines represent the visual discrimination threshold equivalent to 3 JND.

1. **Results – re-analyzing external contrast using smaller leaf litter ROIs**

When estimating contrast between the frogs and their background, the size of the background ROI may affect our estimates of color contrast. For example, by averaging over a larger area of background we may create a mean color that is not itself found in the background. To address this concern, we repeated out analysis of External Contrast with smaller ROIs.

From the center of each of the background images used in the original analysis (n = 60), we selected a frog-size (25 x 25 mm) patch of leaf litter. We then re-computed estimates of chromatic and achromatic contrast between the new background ROIs and the dorsal, front spot, rear spot, and ventral ROIs from all three frog species (*model*, *mimic*, & *cryptic –* N.B. ventral colors were only measured from the model and mimic).

Because the smaller background ROIs increase the possibility for inflated contrast estimates (i.e. the ROI incidentally containing a rare but highly contrasting element, such as a large green leaf), we elected to construct mixed-effects models comparing each frog region to each of the 60 background ROIs. We used the R package *lme4* (Bates et al. 2014) to construct separate models for chromatic and achromatic contrast, for each of our four (dorsal, front spot, rear spot, venter) body regions. This produced eight unique mixed models per visual system. Each model contained a log-transformation of the relevant contrast type as the response variable, with species as a fixed effect and Frog ID and Background ID as random intercepts. For the dorsal body region, which contained three levels, we decomposed significant main effects into pairwise species comparisons using the R package *multcomp* and correcting p values using the single-step method (Hothorn et al. 2008).

We found that the size of the background ROI did not change the main conclusions of our study. Here, in all analyses we find that the *mimic’s* dorsum is more highly contrasting than the *model’s* in both chromatic and achromatic contrast. That *model’s* front spots are more contrasting than the *mimic’s* in chromatic, but not achromatic, contrast, and that in both chromatic and achromatic contrast, the *model’s* rear spots and the model’s venter are more contrasting than those of the *mimic* (Figures S5-S8; Tables S7-S10).

**Table S7.** Results from the additional visual modelling of external contrast using the bird visual model (frog colors vs the frog-sized leaf litter ROIs).

|  | Chromatic Contrast (ΔS) | Achromatic Contrast (ΔL) |
| --- | --- | --- |
| Dorsal | $F_{57}^{2}$ = 55.347, p < 0.001 | $F_{57}^{2}$ = 18.03, p < 0.001 |
| ABI vs AZA | z = -3.70, p < 0.001 | z = -5.58, p < 0.001 |
| ABI vs AHY | z = -6.68, p < 0.001 | z = 0.87, p = 0.659 |
| AZA vs AHY | z = -10.38, p < 0.001 | z = -4.71, p < 0.001 |
| Front Spot  ABI vs AZA | $F_{38}^{1}$ = 104.90, p < 0.001 | $F_{38}^{1}$ = 1.72, p = 0.198 |
| Rear Spot  ABI vs AZA | $F_{38}^{1}$ = 21.16, p < 0.001 | $F_{38}^{1}$ = 70.30, p < 0.001 |
| Venter  ABI vs AZA | $F_{38}^{1}$ = 201.54, p < 0.001 | $F_{38}^{1}$ = 75.11, p < 0.001 |

Note: Treatment codes for species (ABI = *Am. bilinguis*, AZA = *Al. zaparo*, & AHY = *Ad. hylaedactyla*).

**
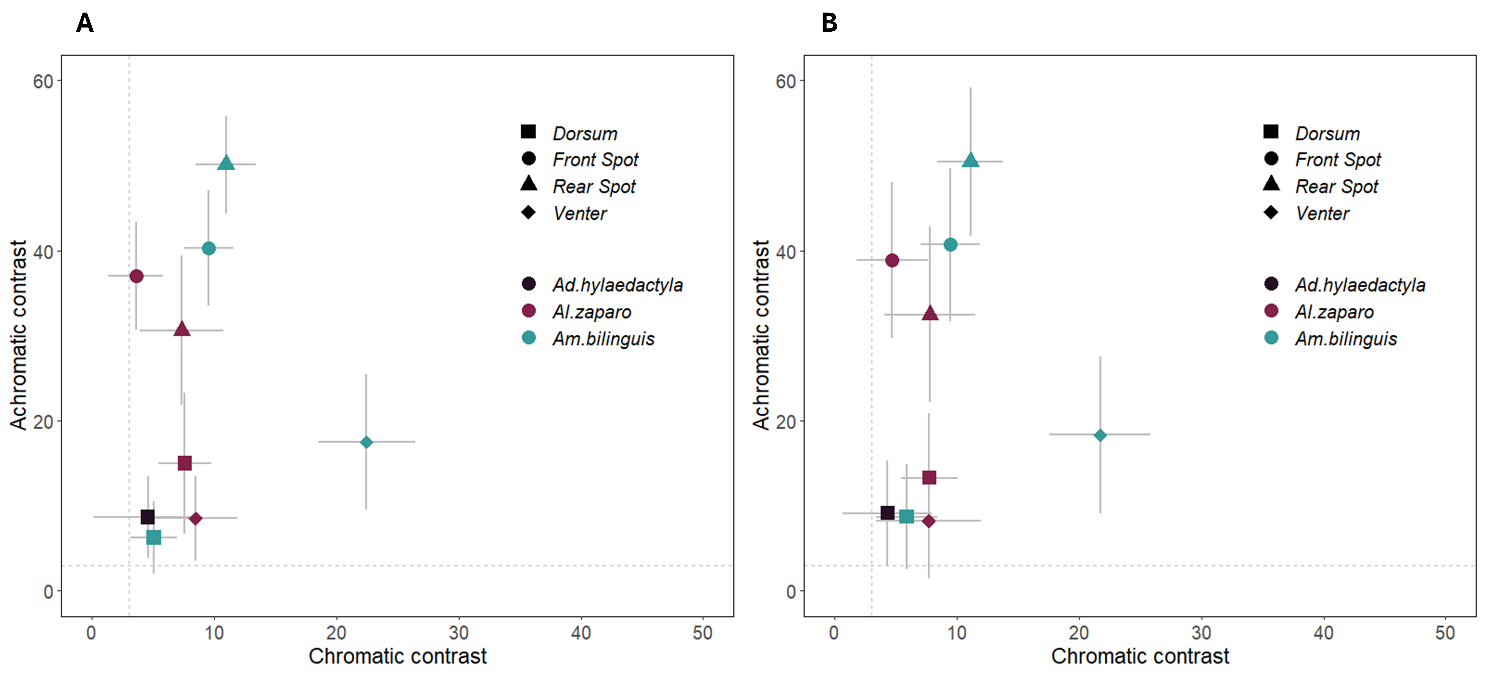
Figure S5: Visual modelling** (bird visual model). Chromatic (ΔS) and achromatic (ΔL) contrast (means ± SD from the raw data), between the colors (square = dorsum, circle = front spot, triangle = rear spot, & diamond = venter) of the frogs (red = *Am. bilinguis*, blue = *Al. zaparo*, & black = *Ad. hylaedactyla*) and the leaf litter background. The grey dotted lines represent the visual discrimination threshold equivalent to 3 JND. A. Original external contrasts computed using whole leaf litter scene as background ROI. B. Alternate contrasts computed using smaller (25 x 25 mm) area as background ROI.

**Table S8.** Results from the additional visual modelling of external contrast using the snake visual model (frog colors vs the frog-sized leaf litter ROIs).

|  | Chromatic Contrast (ΔS) | Achromatic Contrast (ΔL) |
| --- | --- | --- |
| Dorsal | $F_{57}^{2}$ = 3.36, p = 0.042 | $F_{57}^{2}$ = 14.61, p < 0.001 |
| ABI vs AZA | z = -2.55, p = 0.029 | z = -5.27, p < 0.001 |
| ABI vs AHY | z = 1.67, p = 0.217 | z = 1.61, p = 0.240 |
| AZA vs AHY | z = -0.88, p = 0.651 | z = -3.66, p < 0.001 |
| Front Spot  ABI vs AZA | $F_{38}^{1}$ = 73.97, p < 0.001 | $F_{38}^{1}$ = 2.02, p = 0.16 |
| Rear Spot  ABI vs AZA | $F_{38}^{1}$ = 29.26, p < 0.001 | $F_{38}^{1}$ = 74.23, p < 0.001 |
| Venter  ABI vs AZA | $F_{38}^{1}$ = 151.50, p < 0.001 | $F_{38}^{1}$ = 110.66, p < 0.001 |

Note: Treatment codes for species (ABI = *Am. bilinguis*, AZA = *Al. zaparo*, & AHY = *Ad. hylaedactyla*).

**
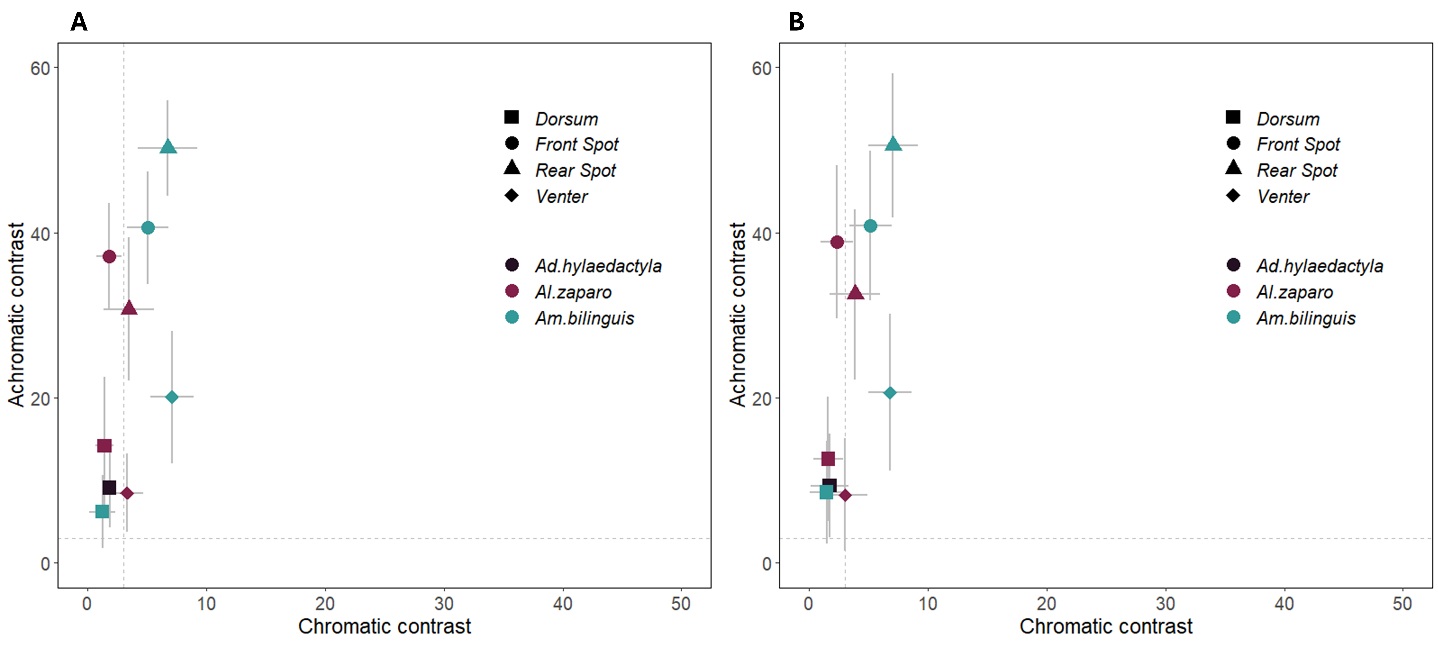
Figure S6: Visual modelling** (snake visual model). Chromatic (ΔS) and achromatic (ΔL) contrast (means ± SD from the raw data), between the colors (square = dorsum, circle = front spot, triangle = rear spot, & diamond = venter) of the frogs (red = *Am. bilinguis*, blue = *Al. zaparo*, & black = Ad*. hylaedactyla*) and the leaf litter background. The grey dotted lines represent the visual discrimination threshold equivalent to 3 JND. A. Original external contrasts computed using whole leaf litter scene as background ROI. B. Alternate contrasts computed using smaller (25 x 25 mm) area as background ROI.

**Table S9.** Results from the additional visual modelling of external contrast using the poison frog visual model (frog colors vs the frog-sized leaf litter ROIs).

|  | Chromatic Contrast (ΔS) | Achromatic Contrast (ΔL) |
| --- | --- | --- |
| Dorsal | $F_{57}^{2}$ = 8.03, p < 0.001 | $F_{57}^{2}$ = 16.61, p < 0.001 |
| ABI vs AZA | z = -3.90, p < 0.001 | z = -5.58, p < 0.001 |
| ABI vs AHY | z = 1.15, p = 0.485 | z = 1.45, p = 0.316 |
| AZA vs AHY | z = -2.75, p = 0.016 | z = -4.11, p < 0.001 |
| Front Spot  ABI vs AZA | $F_{38}^{1}$ = 26.52, p < 0.001 | $F_{38}^{1}$ = 1.93, p = 0.173 |
| Rear Spot  ABI vs AZA | $F_{38}^{1}$ = 12.88, p < 0.001 | $F_{38}^{1}$ = 72.62, p < 0.001 |
| Venter  ABI vs AZA | $F_{38}^{1}$ = 171.51, p < 0.001 | $F_{38}^{1}$ = 89.64, p < 0.001 |

Note: Treatment codes for species (ABI = *Am. bilinguis*, AZA = *Al. zaparo*, & AHY = *Ad. hylaedactyla*).

**
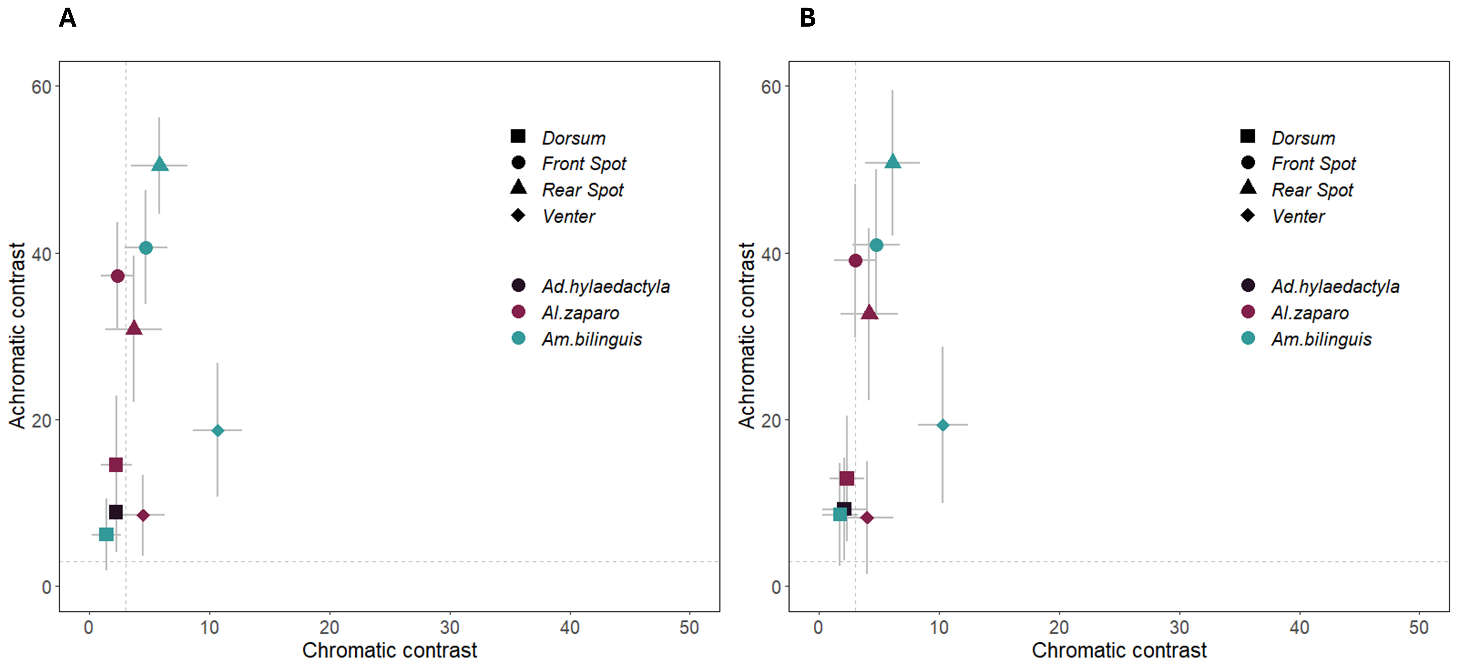
Figure S7: Visual modelling** (poison frog visual model). Chromatic (ΔS) and achromatic (ΔL) contrast (means ± SD from the raw data), between the colors (square = dorsum, circle = front spot, triangle = rear spot, & diamond = venter) of the frogs (red = *Am. bilinguis*, blue = *Al. zaparo*, & black = Ad*. hylaedactyla*) and the leaf litter background. The grey dotted lines represent the visual discrimination threshold equivalent to 3 JND. A. Original external contrasts computed using whole leaf litter scene as background ROI. B. Alternate contrasts computed using smaller (25 x 25 mm) area as background ROI.

**Table S10.** Results from the additional visual modelling of external contrast using the human visual model (frog colors vs the frog-sized leaf litter ROIs).

|  | Chromatic Contrast (ΔS) | Achromatic Contrast (ΔL) |
| --- | --- | --- |
| Dorsal | $F_{57}^{2}$ = 26.00, p < 0.001 | $F_{57}^{2}$ = 17.23, p < 0.001 |
| ABI vs AZA | z = -3.01, p = 0.006 | z = -5.66, p < 0.001 |
| ABI vs AHY | z = -4.13, p < 0.001 | z = 1.46, p = 0.310 |
| AZA vs AHY | z = -7.19, p < 0.001 | z = -4.19, p < 0.001 |
| Front Spot  ABI vs AZA | $F_{38}^{1}$ = 71.73, p < 0.001 | $F_{38}^{1}$ = 3.35, p = 0.075 |
| Rear Spot  ABI vs AZA | $F_{38}^{1}$ = 10.63, p = 0.002 | $F_{38}^{1}$ = 72.75, p < 0.001 |
| Venter  ABI vs AZA | $F_{38}^{1}$ = 183.68, p < 0.001 | $F_{38}^{1}$ = 42.28, p < 0.001 |

Note: Treatment codes for species (ABI = *Am. bilinguis*, AZA = *Al. zaparo*, & AHY = *Ad. hylaedactyla*).

**
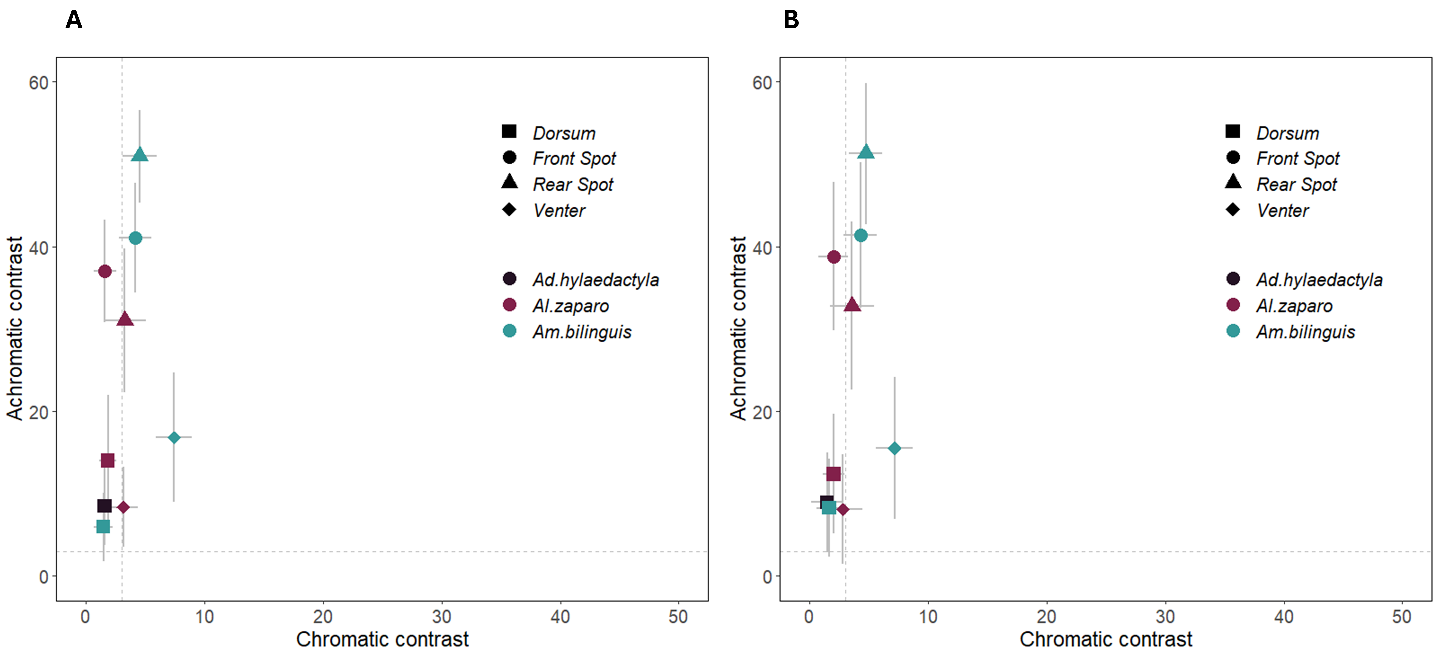
Figure S8: Visual modelling** (human visual model). Chromatic (ΔS) and achromatic (ΔL) contrast (means ± SD from the raw data), between the colors (square = dorsum, circle = front spot, triangle = rear spot, & diamond = venter) of the frogs (red = *Am. bilinguis*, blue = *Al. zaparo*, & black = Ad*. hylaedactyla*) and the leaf litter background. The grey dotted lines represent the visual discrimination threshold equivalent to 3 JND. A. Original external contrasts computed using whole leaf litter scene as background ROI. B. Alternate contrasts computed using smaller (25 x 25 mm) area as background ROI.

1. **References**

Barnett JB, Yeager J, McEwen BL, Kinley I, Anderson HM, Guevara J. 2023 Size-dependent colouration balances conspicuous aposematism and camouflage. *J. Evol. Biol.* **36**, 1010-1019. (doi:10.1111/jeb.14143)

Bates D, Mächler M, Bolker B, Walker S. 2015 Fitting linear mixed-effects models using lme4. *J. Stat. Softw*. **67**, 1-48. (doi:10.18637/jss.v067.i01)

Hart NS, Partridge JC, Cuthill IC, Bennett ATD. 2000 Visual pigments, oil droplets, ocular media and cone photoreceptor distribution in two species of passerine bird: the blue tit (*Parus caeruleus* L.) and the blackbird (*Turdus merula* L.). *J. Comp. Physiol. A* **186**, 375-387. (doi:10.1007/s003590050437)

Hothorn T, Bretz F, Westfall P. 2008 Simultaneous inference in general parametric models. *Biom. J*. **50**, 346-363. (doi:10.1002/bimj.200810425)

Maan ME, Cummings ME. 2012 Poison frog colors are honest signals of toxicity, particularly for bird predators. *Am. Nat.* **179**, E1-E14. (doi:10.1086/663197)

Macedonia JM, Lappin AK, Loew ER, Mcguire JA, Hamilton PS, Plasman M, Brandt Y, Lemos-Espinal JA, Kemp DJ. 2009 Conspicuousness of Dickerson's collared lizard (*Crotaphytus dickersonae*) through the eyes of conspecifics and predators. *Biol. J. Linn. Soc.* **97**, 749-765. (doi:10.1111/j.1095-8312.2009.01217.x)

Maia R, Gruson H, Endler JA, White TE. 2019 pavo 2: New tools for the spectral and spatial analysis of colour in R. *Methods Ecol. Evol.* **10**, 1097-1107. (doi:10.1111/2041-210x.13174)

Siddiqi A, Cronin TW, Loew ER, Vorobyev M, Summers K. 2004 Interspecific and intraspecific views of color signals in the strawberry poison frog *Dendrobates pumilio*. *J. Exp. Biol*. **207**, 2471-2485. (doi:10.1242/jeb.01047)

Smith VC, Pokorny J. 1975 Spectral sensitivity of the foveal cone photopigments between 400 and 500 nm. *Vis. Res*. **15**, 161-171. (doi:10.1016/0042-6989(75)90203-5)

Vorobyev M, Osorio D. 1998 Receptor noise as a determinant of colour thresholds. *Proc. R. Soc. B* **265**, 351-358. (doi:10.1098/rspb.1998.0302)

Yeager J, Barnett JB. 2020 Ultraviolet components offer minimal contrast enhancement to an aposematic signal. *Ecol. Evol.* **10**, 13576-13582. (doi:10.1002/ece3.6969)

Yeager J, Barnett JB. 2021 The influence of ultraviolet reflectance differs between conspicuous aposematic signals in neotropical butterflies and poison frogs. *Ecol. Evol.* **11**, 13633-13640. (doi:10.1002/ece3.7942)
